# Supplementary material for: TLR4-Mediated Pathway Triggers Interferon-Independent G0 Arrest and Antiviral SAMHD1 Activity in Macrophages
Source: Cell Rep. 2020 Mar 24;30(12):3972–3980.e5. doi: 10.1016/j.celrep.2020.03.008 (PMC7109521; doi:10.1016/j.celrep.2020.03.008)
Supplement: Document S1. Figures S1–S4 and Table S1 [file mmc1.pdf]

Cell Reports, Volume *30*

Supplemental Information

TLR4-Mediated Pathway
Triggers Interferon-Independent G0 Arrest
and Antiviral SAMHD1 Activity in Macrophages

Petra Mlcochova, Helena Winstone, Lorena Zuliani-Alvarez, and Ravindra K. Gupta

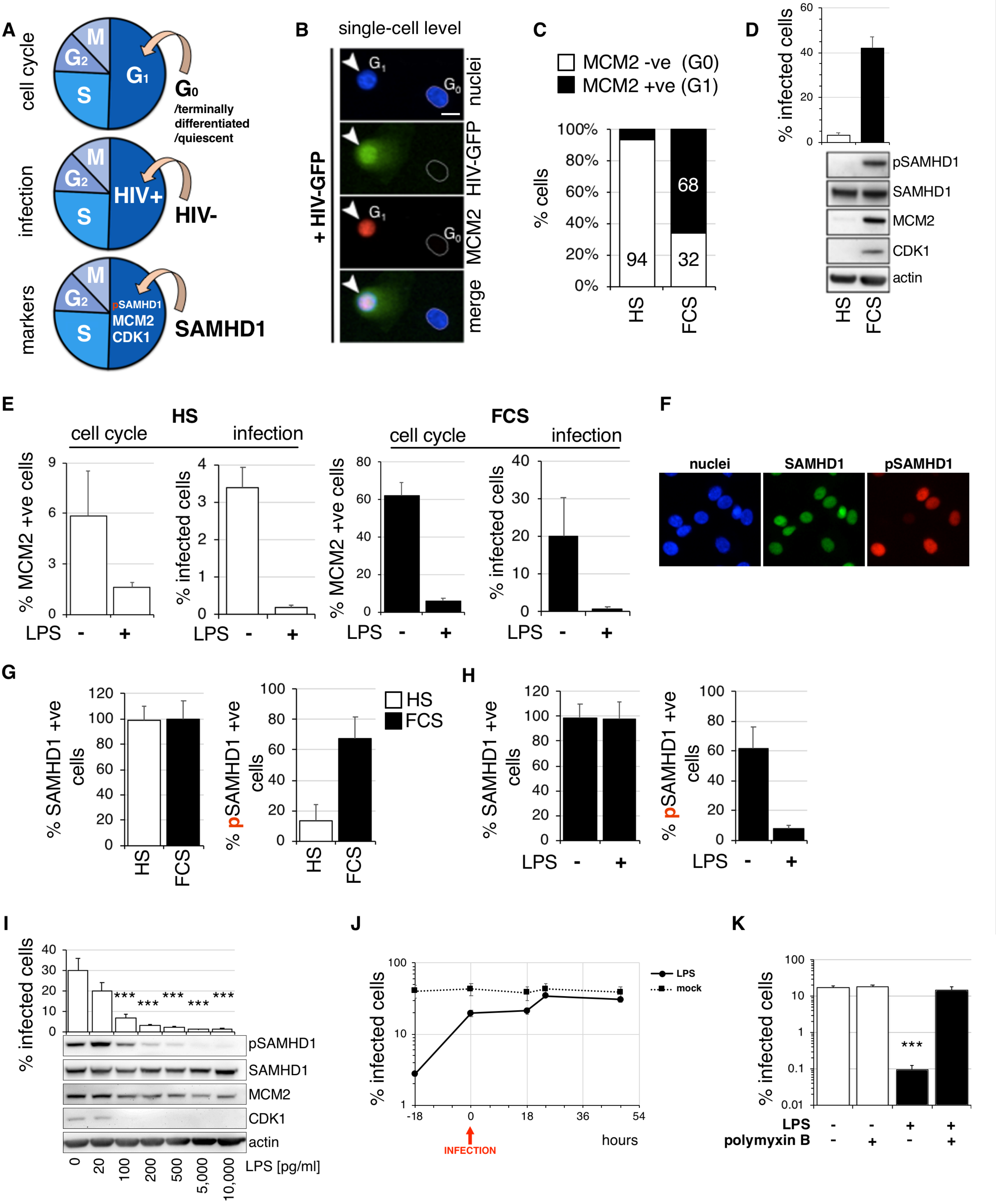

A
cell cycle
infection
markers
M
G2
G1
S
G0
/terminally differentiated /quiescent
HIV+
HIV-
pSAMHD1
MCM2
CDK1
SAMHD1
B
single-cell level
+ HIV-GFP
nuclei
HIV-GFP
MCM2
merge
C
MCM2 -ve (G0)
MCM2 +ve (G1)
% cells
94
32
68
HS
FCS
D
% infected cells
pSAMHD1
SAMHD1
MCM2
CDK1
actin
HS
FCS
E
HS
FCS
cell cycle
infection
% MCM2 +ve cells
% infected cells
LPS - +
F
nuclei
SAMHD1
pSAMHD1
G
% SAMHD1 +ve cells
% pSAMHD1 +ve cells
HS
FCS
H
% SAMHD1 +ve cells
% pSAMHD1 +ve cells
LPS - +
I
% infected cells
***
pSAMHD1
SAMHD1
MCM2
CDK1
actin
0
20
100
200
500
5,000
10,000
LPS [pg/ml]
J
% infected cells
LPS
mock
hours
INFECTION
K
% infected cells
***
LPS - - + +
polymyxin B - + - +

Figure S1: HS and FCS differentiation protocol used to study TLR4 mediated block to HIV-1 infection. Related to Figure 1-4. **(A)** Diagram of G_0 to G_1 transition in MDM based on our previous study in EMBO J (Mlcochova et al., 2017). MDM in G_0 are quiescent cells where SAMHD1 is dephosphorylated/active. G_0 cells are not permissive to HIV-1 infection. MDM after transition into G_1 phase are positive for MCM2 and CDK1, markers of cell cycle re-entry and G_1 phase. SAMHD1 is phosphorylated/inactive in G_1 cells and MDM are permissive to HIV-1 infection. **(B)** Example of single-cell analysis showing MDM in G_0 (negative for MCM2, non-infected) and G_1 phase (positive for MCM2, HIV-1 infected). In our experiments on average 10^4 cells are recorded and analysed using automated cell-imaging system Hermes WiScan and ImageJ. Scale bars, 20µm. **(C,D)** Quantification of cells in G_0 and G_1 phase. By simple culturing condition we can keep MDM in G_0 (by using Human Serum, HS) or induce G_0 to G_1 transition (by using Fetal Calf Serum, FCS). Quantification can be achieved at single cell level **(B,C)** or from cell lysates using immunoblot **(D)**. **(E)** Cells cultured in HS (MDM mostly in G_0) or FCS (MDM mostly in G_1) were treated with LPS and infected by VSV-G pseudotyped HIV-1 18h later. The percentage of infected and MCM2 positive cells were determined 48h post-infection. ($n = 3$, mean ± s.e.m.; ***P-value≤0.001, **P-value ≤ 0.01, paired t-test). **(F-H)** Detection of pSAMHD1 at single-cell level. In our experiments on average 10^4 cells are recorded and analysed using automated cell-imaging system Hermes WiScan and ImageJ. **(F)** Representative example of immunofluorescence staining of MDM for total SAMHD1 and phosphorylated SAMHD1. Scale bars, 20µm. **(G)** Quantification of SAMHD1 and pSAMHD1 expression in HS and FCS cultured macrophages. **(H)** Quantification of SAMHD1 and pSAMHD1 expression in FCS cultured macrophages 18h after LPS treatment. **(I-K)** Cells were infected by VSV-G pseudotyped HIV-1 and the percentage of infected cells was determined 48h post-infection. (n=3, mean±SEM; ***P-value≤0.001, paired t-test). **(I)** MDM were treated with increasing concentration of LPS 18h before infection. Cells from a representative donor were used for immunoblotting. **(J)** MDM were treated with LPS at different timepoints starting 18h before infection, at the time of infection and after infection. **(K)** MDM were treated with LPS +/- polymyxin B that blocks the biological effect of LPS.

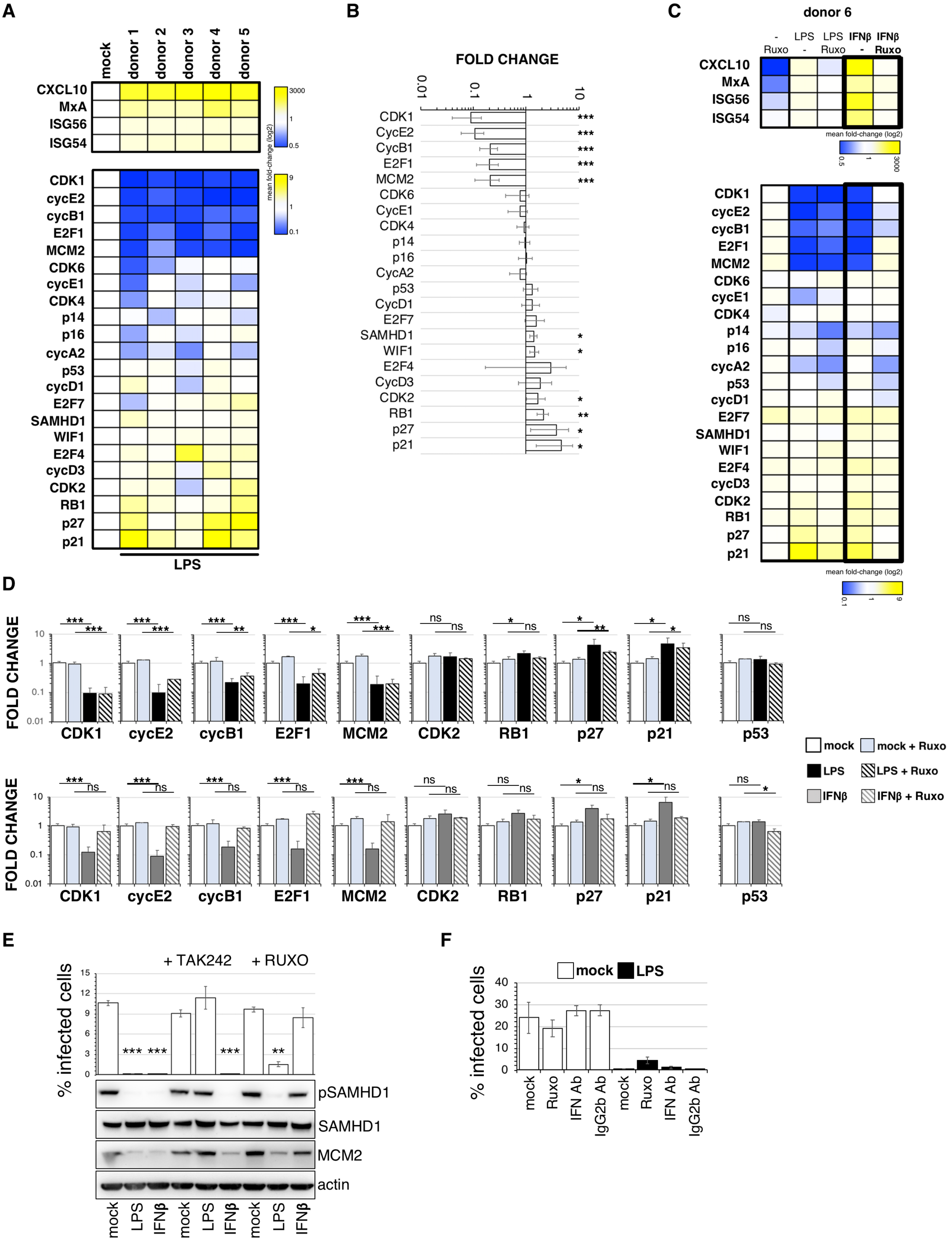

A
mock
donor 1
donor 2
donor 3
donor 4
donor 5
CXCL10
MxA
ISG56
ISG54
3000
1
0.5
mean fold-change (log2)
9
1
0.1
CDK1
cycE2
cycB1
E2F1
MCM2
CDK6
cycE1
CDK4
p14
p16
cycA2
p53
cycD1
E2F7
SAMHD1
WIF1
E2F4
cycD3
CDK2
RB1
p27
p21
LPS
B
FOLD CHANGE
0.01
0.1
1
10
CDK1 ***
CycE2 ***
CycB1 ***
E2F1 ***
MCM2 ***
CDK6
CycE1
CDK4
p14
p16
CycA2
p53
CycD1
E2F7
SAMHD1 *
WIF1 *
E2F4
CycD3
CDK2 *
RB1 **
p27 *
p21 *
C
donor 6
- Ruxo
LPS -
LPS Ruxo
IFNβ -
IFNβ Ruxo
CXCL10
MxA
ISG56
ISG54
mean fold-change (log2)
0.5
1
3000
CDK1
cycE2
cycB1
E2F1
MCM2
CDK6
cycE1
CDK4
p14
p16
cycA2
p53
cycD1
E2F7
SAMHD1
WIF1
E2F4
cycD3
CDK2
RB1
p27
p21
mean fold-change (log2)
0.1
1
9
D
FOLD CHANGE
10
1
0.1
0.01
CDK1
cycE2
cycB1
E2F1
MCM2
CDK2
RB1
p27
p21
p53
***
**
*
ns
mock
mock + Ruxo
LPS
LPS + Ruxo
IFNβ
IFNβ + Ruxo
E
+ TAK242
+ RUXO
% infected cells
15
12
9
6
3
0
***
***
***
**
pSAMHD1
SAMHD1
MCM2
actin
mock
LPS
IFNβ
mock
LPS
IFNβ
mock
LPS
IFNβ
F
mock
LPS
% infected cells
40
30
20
10
0
mock
Ruxo
IFN Ab
IgG2b Ab
mock
Ruxo
IFN Ab
IgG2b Ab

Figure S2: IFN-independent and dependent G_0 arrest in human MDM. Related to Figure 3. (**A)** A heat map depicting differential gene expression patterns of cell cycle associated transcripts in MDM from 5 different donors treated with LPS for 18h. The colour scale bar corresponds to log-fold expression. (**B)** Relative expression levels (fold change) of cell cycle associated transcripts. (*n* = 6, mean ± SEM; ***P-value≤*0.001,* ***P*-value ≤ 0.01, **P*-value ≤ 0.1, paired *t*-test). (**C)** A heat map depicting differential gene expression patterns of cell cycle associated transcripts in MDM treated with LPS or IFN**β** in the presence or absence of RUXO. The colour scale bar corresponds to log-fold expression. **(D)** Relative expression levels (fold changes) of statistically significantly changed cell cycle associated transcripts after LPS or IFN**β** treatment in the presence or absence of RUXO. (*n* = 4, mean ± s.e.m.; ***P-value≤*0.001,* ***P*-value ≤ 0.01, **P*-value ≤ 0.1, (ns) non-significant, paired *t*-test). **(E)** MDM were treated with TAK242 and RUXO 6h before addition of LPS and interferon b (IFN**β**). Cells were infected by VSV-G pseudotyped HIV-1 18h later. The percentage of infected cells was determined 48h post-infection. (*n* = 3, mean ± s.e.m.; ***P-value≤*0.001,* ***P*-value ≤ 0.01, paired *t*-test). Cells from a representative donor were used for immunoblotting. (**F)** MDM were exposed to anti-IFN Ab/IgG2b non-specific Ab and treated with LPS. Cells were infected by VSV-G pseudotyped HIV-1 18h later. The percentage of infected cells was determined 48h post-infection. (*n* = 3, mean ± s.e.m.; ****P*-value ≤ 0.01, paired *t*-test).

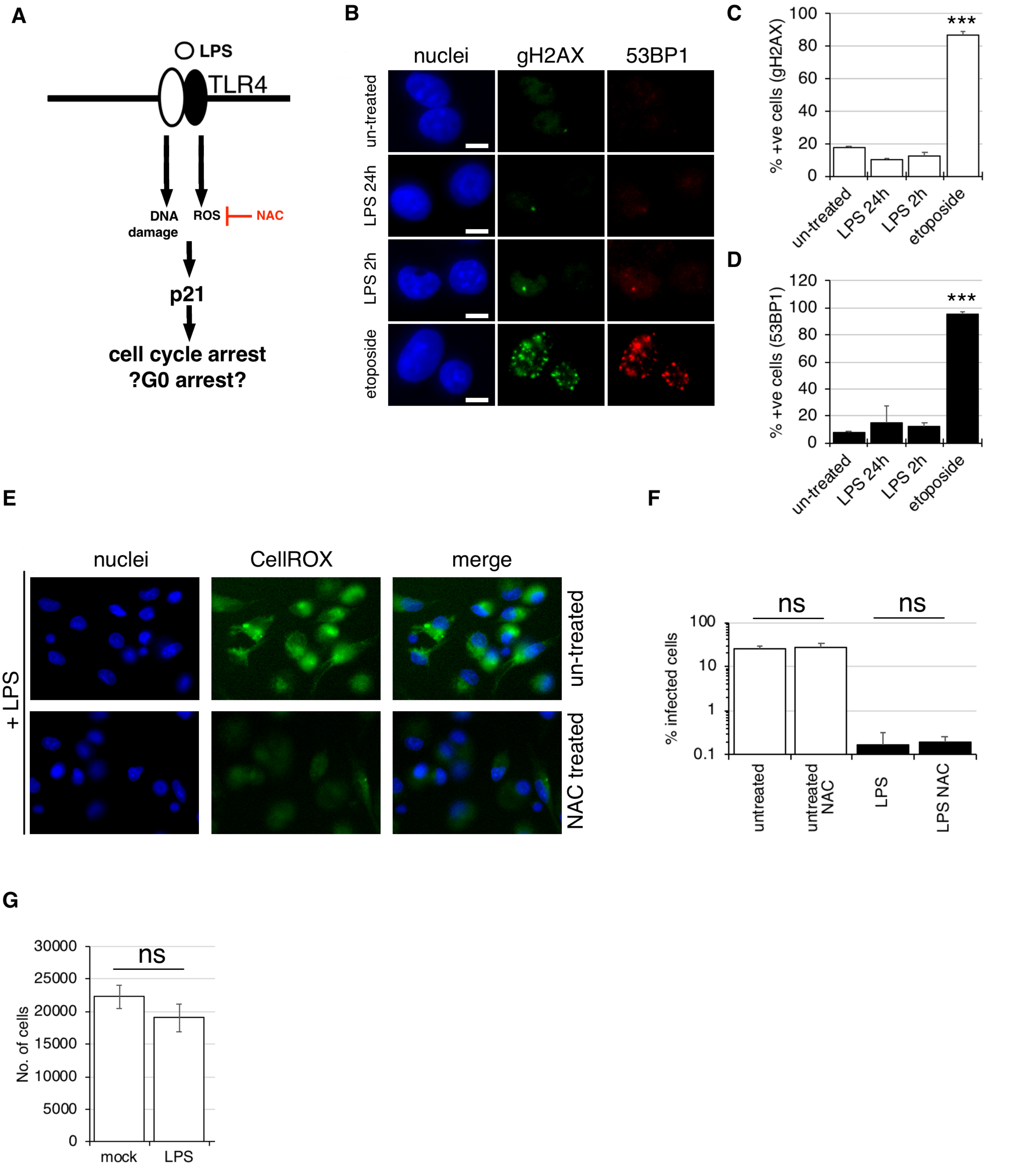

Figure S3: DNA damage and ROS during LPS mediated TLR4 activation in human macrophages. Related to Figure 3. (A) Simplified diagram of LPS activation triggering oxidative stress (ROS production) and DNA damage. ROS inhibitor N-acetyl-L-cysteine (NAC). **(B)** MDM were treated with LPS for 2 or 24h, or with Etoposide for 2h (positive control for DNA damage). Cells were fixed and stained for DNA damage foci positive for H2AX and 53BP1. **(C,D)** Quantification of **(C)** H2AX or **(D)** 53BP1 positive cells. 1,000 cells were acquired using Hermes WiScan automated cell-imaging system (IDEA Bio-Medical Ltd. Rehovot, Israel) and analysed using MetaMorph and ImageJ software. ($n = 3$, mean ± SEM). ***P-value ≤ 0.01, paired *t*-test. **(E)** MDM were treated with LPS in the presence or absence of ROS inhibitor NAC and labelled with CellROX to detect ROS. **(F)** MDM were treated with LPS in the presence or absence of ROS inhibitor NAC 18h before infection. Cells were infected by VSV-G pseudotyped HIV-1 and % infected cells were determined 48h post-infection. ($n = 3$, mean ± SEM). (ns) non-significant, paired *t*-test. **(G)** MDM treated or untreated with LPS were stained for nuclei using DAPI stain. Cell numbers were quantified using Hermes WiScan automated cell-imaging system (IDEA Bio-Medical Ltd. Rehovot, Israel), MetaMorph and ImageJ software ($n = 7$, mean ± SEM). (ns) non-significant, paired *t*-test.

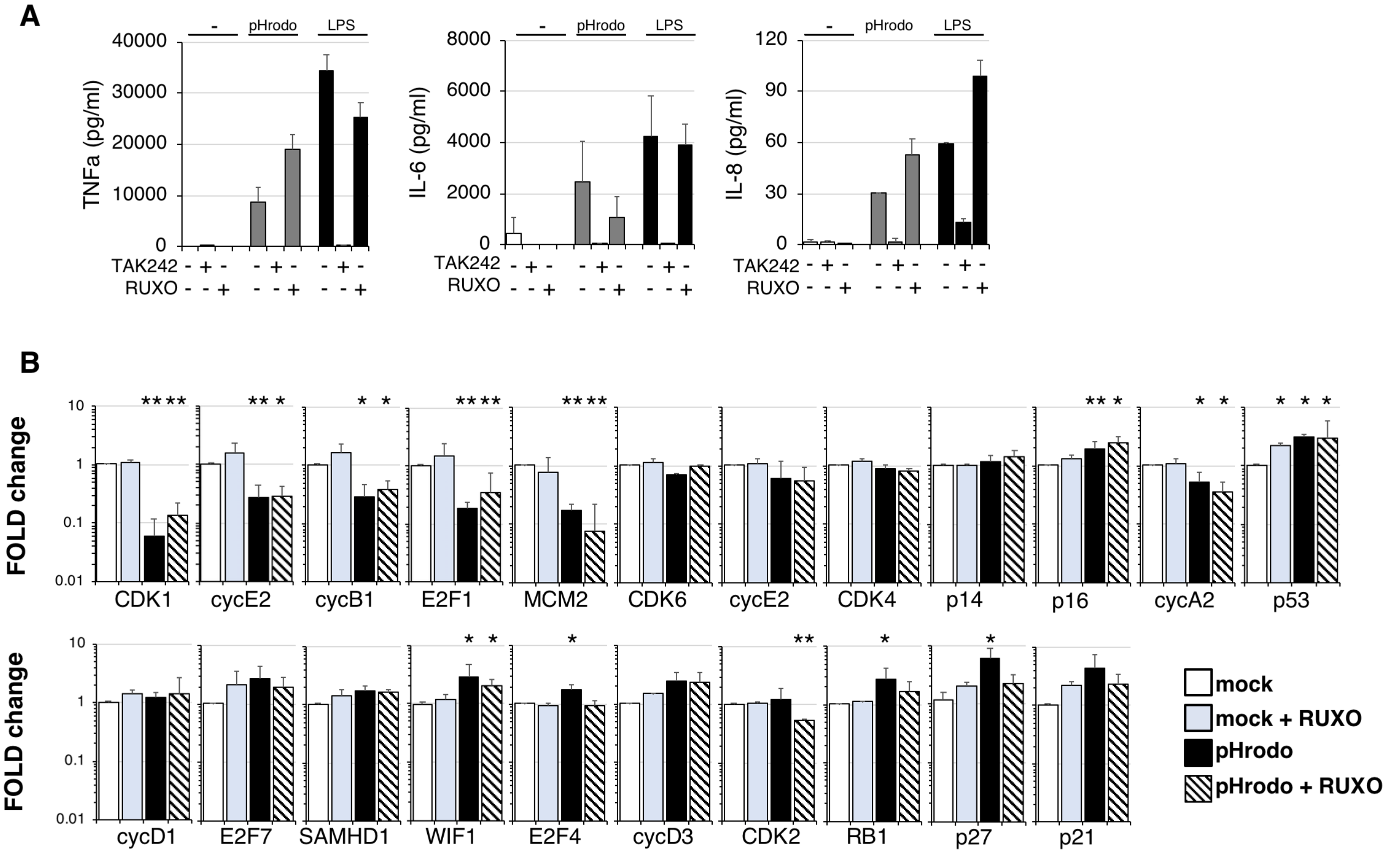

Figure S4: TLR4 activation by gram-negative bacteria. Related to Figure 4. A) MDM were treated with pHrodo E.coli or LPS in the presence or absence of RUXO and cytokines were measured in culture media 24h later. **B)** Relative expression levels (fold changes) of full panel of cell cycle associated transcripts. MDM were treated with RUXO 6h before addition of pHrodo E.coli. Cells were collected 24h later. (n = 3, mean ± SEM). **P-value ≤ 0.01, *P-value ≤ 0.1, paired t-test.
