# Supplementary material for: TLR4-Mediated Pathway Triggers Interferon-Independent G0 Arrest and Antiviral SAMHD1 Activity in Macrophages
Source: Cell Rep. 2020 Mar 24;30(12):3972–3980.e5. doi: 10.1016/j.celrep.2020.03.008 (PMC7109521; doi:10.1016/j.celrep.2020.03.008)
Supplement: Document S2. Article plus Supplemental Information [file mmc2.pdf]

Report

Cell Reports

TLR4-Mediated Pathway Triggers Interferon-Independent G0 Arrest and Antiviral SAMHD1 Activity in Macrophages

Graphical Abstract

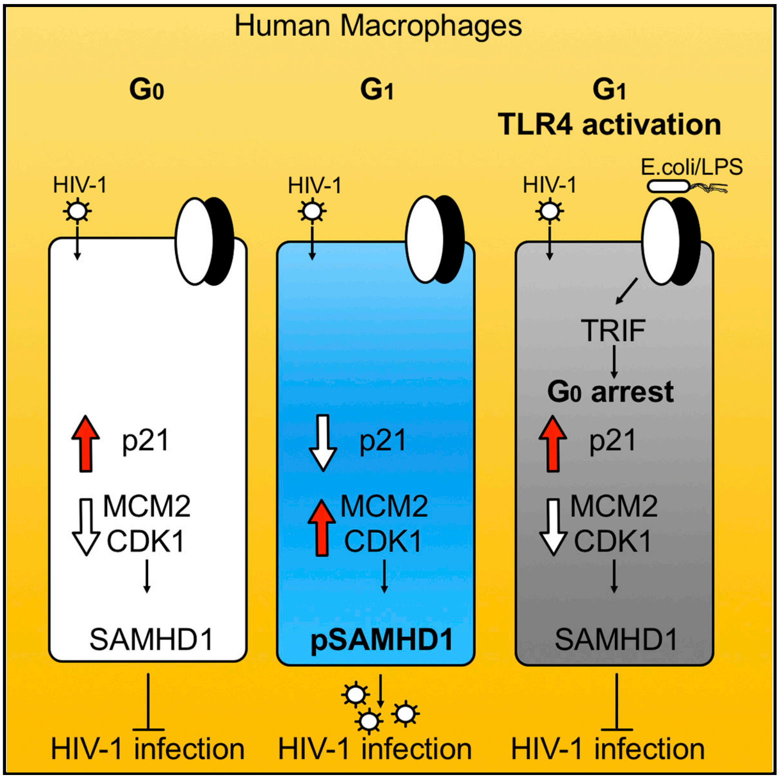

Highlights

- TLR4 activation mediates TRIF-dependent G0 arrest in human primary macrophages
- G0 arrest regulates phosphorylation and antiretroviral activity of SAMHD1
- SAMHD1 is responsible for the IFN-independent HIV-1 blockade after TLR4 activation
- Whole gram-negative bacteria induce IFN-independent G0 arrest in human MDMs

Authors

Petra Mlcochova, Helena Winstone, Lorena Zuliani-Alvarez, Ravindra K. Gupta

Correspondence

rkg20@cam.ac.uk

In Brief

Mlcochova et al. demonstrate that TLR4 activation regulates the cell cycle in human primary macrophages. This culminates in G0 arrest and activation of the antiviral protein SAMHD1, suggesting that macrophages can achieve a heightened state of alert in response to pathogen-associated danger signals in macrophages prior to type I IFN secretion.

Mlcochova et al., 2020, Cell Reports *30*, 3972–3980
March 24, 2020 © 2020 The Author(s).
https://doi.org/10.1016/j.celrep.2020.03.008

OPEN ACCESS
CellPress

Cell Reports
Report

TLR4-Mediated Pathway Triggers Interferon-Independent G0 Arrest and Antiviral SAMHD1 Activity in Macrophages

Petra Mlcochova,[1] Helena Winstone,[2] Lorena Zuliani-Alvarez,[2] and Ravindra K. Gupta[1,3,4,*]
[1]Department of Medicine, University of Cambridge, Cambridge, UK
[2]Division of Infection and Immunity, UCL, London, UK
[3]Africa Health Research Institute, Durban, KwaZulu Natal, South Africa
[4]Lead Contact
*Correspondence: rkg20@cam.ac.uk
https://doi.org/10.1016/j.celrep.2020.03.008

SUMMARY

Macrophages exist predominantly in two distinct states, G0 and a G1-like state that is accompanied by phosphorylation of SAMHD1 at T592. Here, we demonstrate that Toll-like receptor 4 (TLR4) activation can potently induce G0 arrest and SAMHD1 antiretroviral activity by an interferon (IFN)-independent pathway. This pathway requires TLR4 engagement with TRIF, but not involvement of TBK1 or IRF3. Exclusive Myd88 activators are unable to trigger G0 arrest or SAMHD1 dephosphorylation, demonstrating this arrest is also Myd88/nuclear factor κB (NF-κB) independent. The G0 arrest is accompanied by p21 upregulation and CDK1 depletion, consistent with the observed SAMHD1 dephosphorylation at T592. Furthermore, we show by SAMHD1 knockdown that the TLR4-activated pathway potently blocks HIV-1 infection in macrophages specifically via SAMHD1. Together, these data demonstrate that macrophages can mobilize an intrinsic cell arrest and anti-viral state by activating TLR4 prior to IFN secretion, thereby highlighting the importance of cell-cycle regulation as a response to pathogen-associated danger signals in macrophages.

INTRODUCTION

Macrophages are the first line of defense against invading pathogens, sensing through pathogen recognition receptors (PRRs) and initiating innate and adaptive responses. The most studied PRRs are Toll-like receptors (TLRs), expressed in monocytes, macrophages, and dendritic cells. They play a fundamental role in recognition of pathogen-associated molecular patterns expressed on infectious agents and subsequently initiate a series of inflammatory events that depend upon the MyD88 and/or TRIF signaling pathways (Iwasaki and Medzhitov, 2004). The MyD88-dependent pathway is activated by all TLRs except TLR3 that signals only through TRIF. TLR4, however, activates both MyD88- and TRIF-dependent pathways in response to lipopolysaccharide (LPS), a component from the wall of gram-negative bacteria (Park and Lee, 2013); LPS-induced TLR4/TRIF-dependent signaling results in TBK1 activation, translocation of IRF3 into the nucleus, and type I IFN production, whereas MyD88-dependent signaling results in nuclear factor κB (NF-κB) translocation and expression of various pro-inflammatory genes (Akira and Takeda, 2004; Kawai et al., 2001).

Macrophages, cells normally residing in a G0/terminally differentiated state, can re-enter the cell cycle into a G1-like phase, expressing certain cellular cell-cycle factors (Mlcochova et al., 2017, 2018), including cyclin-dependent kinase 1 (CDK1). This kinase is known to phosphorylate and deactivate the antiviral activity of SAMHD1 (Cribier et al., 2013; White et al., 2013), a deoxynucleotide-triphosphate (dNTP) hydrolase that restricts HIV-1 (Arnold et al., 2015; Goldstone et al., 2011; Hrecka et al., 2011; Laguette et al., 2011; Lahouassa et al., 2012). SAMHD1 phosphorylation at position T592 has been shown to be mediated by CDK1/2 (Cribier et al., 2013; White et al., 2013). Some argue that SAMHD1 phosphorylation at position T592 impairs its dNTP hydrolase activity and allows viral DNA synthesis to occur (Arnold et al., 2015), whereas others propose that T592 phosphorylation does not regulate SAMHD1 dNTPase activity and/or that T592 phosphorylation impacts dNTPase-independent restriction mechanisms (Bhattacharya et al., 2016; Herrmann et al., 2018; Valle-Casuso et al., 2017; Welbourn and Strebel, 2016; White et al., 2013).

Type I interferons (IFNs) are known to arrest cycling cells (Xaus et al., 1999; Zhang et al., 2007) and lead to dephosphorylation/activation of SAMHD1 in monocyte-derived macrophages (MDMs) (Cribier et al., 2013; Szaniawski et al., 2018), although the mechanism is unclear. LPS has known potent inhibitory activity against HIV-1 infection (Bernstein et al., 1991; Franchin et al., 2000; Geonnotti et al., 2010; Kornbluth et al., 1989; Reinhard et al., 2014; Schlaepfer et al., 2014; Verani et al., 2002), partly due to downregulation of receptors for HIV-1 entry and impairment of early steps of the viral life cycle (Franchin et al., 2000; Verani et al., 1997; Wang et al., 2008). The mediators of HIV-1 suppression by LPS-stimulated MDMs are mostly secreted β-chemokines and IFNs (Geonnotti et al., 2010; Schlaepfer et al., 2014; Verani et al., 1997, 2002). However, some data suggest that IFN release by LPS-stimulated macrophages/dendritic cells might not be the main mediator of HIV-1 suppression (Reinhard et al., 2014; Verani et al., 2002), although the underlying mechanism has not been elucidated.

Here we show that TLR4 activation by LPS and whole bacteria can regulate transition between G1 and G0 and SAMHD1 antiviral

This is an open access article under the CC BY license (http://creativecommons.org/licenses/by/4.0/).

activity via a Myd88- and IFN-independent pathway. We show that the G1-to-G0 transition is downstream of TRIF but completely independent from TBK1 and IRF3 activation. The resulting G0 arrest is accompanied by p21 upregulation and SAMHD1 dephosphorylation. This demonstrates that TLR4 activation can directly induce G0 arrest in human macrophages independent of IFN secretion, while activating a pathogen defense program mediated by SAMHD1. This work adds to growing evidence that G0 arrest is a conserved and important response to danger signals even in cells classically viewed as being terminally differentiated.

RESULTS

TBK1- and IFN-Independent G0 Arrest following TLR4 Engagement by LPS Activates SAMHD1 Antiretroviral Activity

We have shown previously that macrophage transition from G0 to a G1-like state is accompanied by an increase in certain cell-cycle-associated proteins, such as MCM2 and CDK1, as well as phosphorylation of SAMHD1 at T592 that confers increased susceptibility to HIV-1 infection (Mlcochova et al., 2017, 2018). Although the cell-cycle state of macrophages *in vivo* is poorly defined, we demonstrated that two widely accepted *in vitro* MDM differentiation protocols that differ in what type of culture media is used can lead to differences in the proportion of MDMs in G1 (Figure S1). MDMs differentiated in human serum (HS) are mainly in G0 (~94%), and those differentiated in fetal calf serum (FCS) are predominantly in G1 (~70%) (Figure S1C), whereas hierarchical clustering of whole genome expression data showed that HS- and FCS-cultured MDMs cluster together and are distinct from closely related myeloid cells (Mlcochova et al., 2017). Further, in case of HS differentiation (mostly G0 MDMs), SAMHD1 is dephosphorylated/active. In case of FCS differentiation (MDMs mostly in G1), SAMHD1 is phosphorylated/inactive (Figures S1A and S1F–S1H). In the present study, we used the FCS differentiation protocol (Figure S1).

Treatment with 10 ng/mL LPS for 18 h resulted in a decrease of MCM2 and CDK1 expression and as expected, SAMHD1 dephosphorylation at T592 (Figures 1A and S1I–S1K). These results suggest that LPS treatment led to G1-to-G0 transition (~G0 arrest) in macrophages where SAMHD1 is activated and can block HIV-1 infection as shown in Figure 1A. Of note, HS-differentiated macrophages behaved in a similar way following LPS exposure (Figure S1E). Crucially, the TLR4 inhibitor TAK242 completely prevented G0 arrest and SAMHD1 phosphorylation changes, restoring HIV-1 infection (Figures 1A–1C). Blocking IFN signaling in macrophages by treatment with the JAK1/2 inhibitor ruxolitinib (RUXO) could not prevent G1-to-G0 transition, demonstrating that LPS-induced G0 arrest and SAMHD1 dephosphorylation/activation in human macrophages via TLR4 is IFN independent.

We next used the TBK1 inhibitor BX795 that blocks phosphorylation, nuclear translocation, and transcriptional activity of IRF3. This drug successfully prevented IRF3 translocation to the nucleus (Figure 1C), but could not prevent G1-to-G0 transitioning, SAMHD1 dephosphorylation, and blockade of HIV-1 infection (Figures 1A–1C). Similarly, TBK1 knockdown (KD) was unable to prevent G1-to-G0 transitioning (Figures 1D–1H). By contrast, depletion of TRIF did prevent G1-to-G0 transitioning (Figures 1D–1H), revealing that G0 arrest following TLR4 activation is downstream of TRIF but upstream of TBK1 signaling (Figures 1B and 1D–1H).

MyD88 Activation via TLR4 or TLR5 Does Not Induce G0 Arrest

Tenascin-C (TNC) is an extracellular matrix protein rapidly induced at the site of infection or injury, where it triggers inflammation by activating TLR4 in different cells, including macrophages (Midwood et al., 2016). This TLR4 activation is specifically mediated through the MyD88-dependent pathway (Midwood et al., 2009) (Figure 2A). Flagellin (FLA) is the main component of bacterial flagellum. It binds to TLR5 and induces MyD88-dependent signaling. Both TNC and FLA effectively activated the MyD88-dependent pathway in MDMs, which was confirmed by detectable translocation of NF-κB and production of cytokines (interleukin-6 [IL-6], IL-8) into culture media, but absence of nuclear IRF3 and CXCL10 production (Figures 2B and 2C). Importantly, when MDMs were treated with TNC or FLA, no changes to cell-cycle protein expression, SAMHD1 phosphorylation, or HIV-1 inhibition were detected (Figure 2D). From these experiments we conclude that MyD88 activation is insufficient to induce G0 arrest, dephosphorylation of SAMHD1, and blockade of HIV-1 infection.

LPS Activation of TLR4 Results in IFN-Independent Upregulation of p21

We hypothesized that the LPS-induced IFN-independent G0 arrest would be regulated by expression of negative cell-cycle regulators, such as p16, p21, or p27. Immunoblots performed in the presence of RUXO to block IFN-dependent signaling confirmed that the decrease in CDK1 and MCM2 after LPS treatment was accompanied by increased p21 levels (Figure 3A). Because we were unable to detect p27 or p16 expression in immunoblot, we next sought to further characterize the cell-cycle program changes triggered by LPS in MDMs, using a panel of cell-cycle-associated transcripts measured by qPCR (Figures 3B and S2A–S2D). Statistically significant decreases compared with the untreated control (set to 1) were observed in the following transcripts associated with cell-cycle progression: CDK1; MCM2; and cyclins E2, B1, E1, A2, and E2F1. A significant increase was observed for p21 transcript associated with cell-cycle arrest (Figure 3C, far right panel). The full panel of transcripts is shown in Figures S2A–S2D. It has been suggested that production of reactive oxygen species (ROS) or DNA damage during LPS treatment can activate p21 pathway and cell-cycle arrest in proliferating cells (Cheng et al., 2015; Mytych et al., 2017). In our study, exposure to LPS led to increased p21 expression, but not to markers associated with DNA damage, such as γH2AX and 53BP1 (Figures S3A–S3D). LPS triggered ROS production (Figure S3E) that could be inhibited by using *N*-acetyl-cysteine (NAC). Nevertheless, this inhibition had no effect on LPS-mediated G0 arrest (Figure S3F). Our data thus suggest that ROS or DNA damage is unlikely to be responsible for G0 arrest in human MDMs.

Redundancy of Pathways Activated by TLR4 that Lead to G0 Arrest

IFN as a cause of cell-cycle arrest has been reported in myeloid cells from mice and murine cell lines, as well as in human cell lines (Dey et al., 2000; Xaus et al., 1999; Zhang et al., 2007), monocytes,

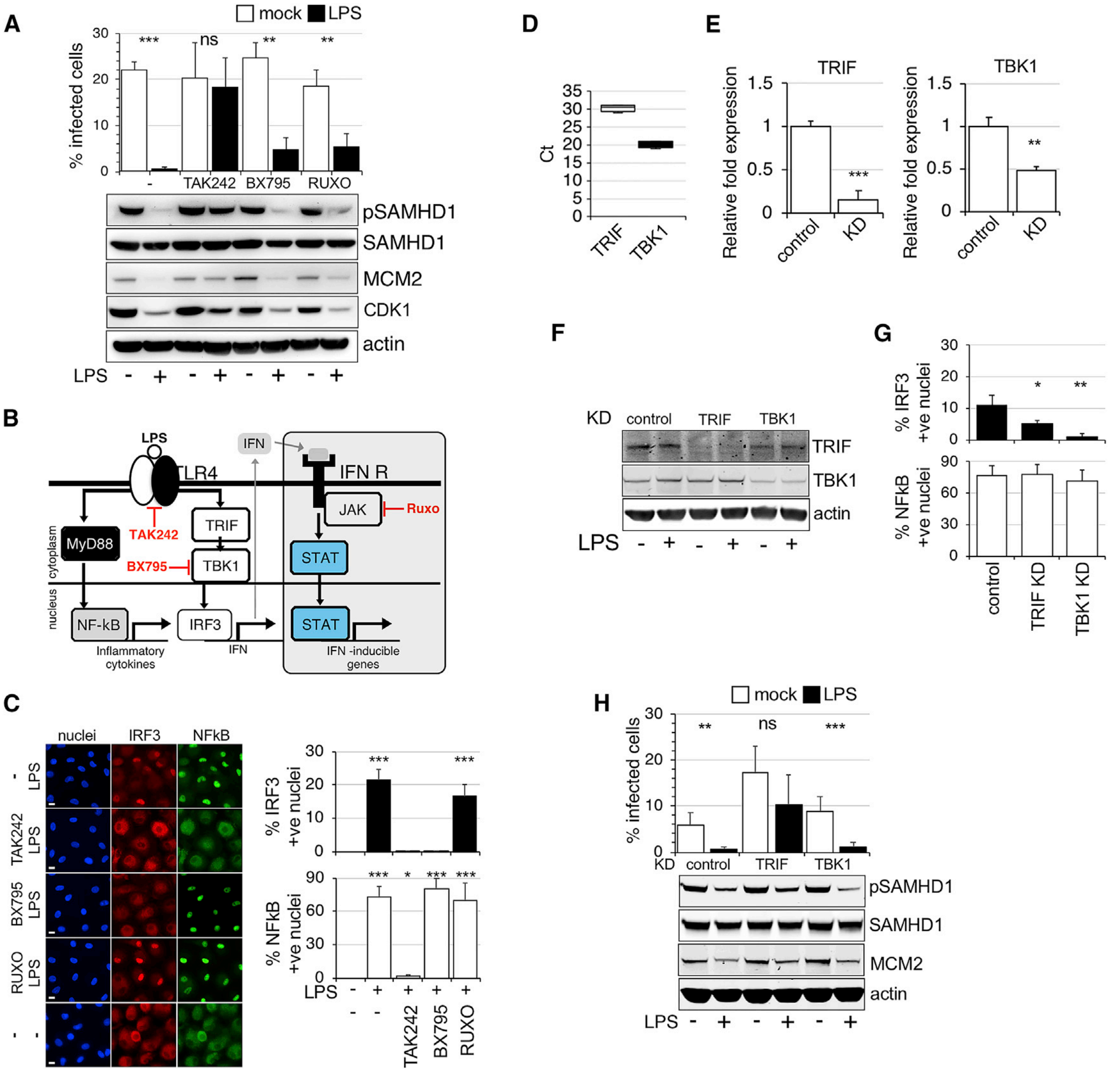

Figure 1. TLR4 Activation Induces G0 Arrest, Dephosphorylates SAMHD1, and Blocks HIV-1 Infection in an Interferon-Independent Manner

(A) MDMs were treated with TAK242, BX795, and RUXO 6 h before addition of LPS. Cells were infected by vesicular stomatitis virus G protein (VSV-G)-pseudotyped HIV-1 18 h later. The percentage of infected cells was determined 48 h post-infection (n = 5, mean ± SEM). Cells from a representative donor were used for immunoblotting.

(B) A simplified diagram of TLR4 signaling in response to LPS. LPS activates both MyD88-dependent and -independent signaling pathways. BX795, an inhibitor of TBK1; RUXO (ruxolitinib), an inhibitor of JAK1/2 kinase that suppresses IFN signaling; TAK242, an inhibitor of TLR4 signaling.

(C) IRF3/NF-κB nuclear translocation assay. Cells were exposed to LPS in the absence or presence of TAK242, BX795, and RUXO, and 2 h later stained for IRF3/NF-κB. The percentage of cells with nuclear staining was determined (n = 3, mean ± SEM). Scale bars, 20 μm.

(D) Expression data of TRIF and TBK1 in MDMs, displayed as cycle threshold (Ct) values (n = 3, mean ± SEM).

(E–H) MDMs were transfected with control or TRIF, TBK1 siRNA. mRNA expression is shown as fold change relative to control (n = 3, mean ± SEM) (E). Cells from a representative donor were used for immunoblotting (F). Cells were exposed to LPS in control or KD cells, and 2 h later stained for IRF3/NF-κB. % of cells with nuclear staining was determined (n = 3, mean ± SEM) (G). MDMs transfected with control or TRIF, TBK1 siRNA were treated with LPS. Cells were infected by VSV-G-pseudotyped HIV-1 18 h later. The percentage of infected cells was determined 48 h post-infection (n = 3 donors, mean ± SEM). Cells from a representative donor were used for immunoblotting (H).

$^{***}p \leq 0.001$; $^{**}p \leq 0.01$; $^{*}p \leq 0.1$; ^{ns}p, non-significant, paired t test.

and T cells (Munn et al., 1996). We next examined the effect of blocking IFN signaling after TLR4 activation, as well as after addition of exogenous IFNβ (Figures S2C–S2F). RUXO, an inhibitor of JAK kinases and IFN signaling, was used to treat MDM 6 h before addition of LPS or IFNβ. As expected, RUXO completely blocked expression of selected interferon stimulated genes (ISGs), CXCL10, MxA, ISG 54, and ISG 56, after both LPS and IFNβ treatment, confirming that IFN signaling is inhibited in both conditions (Figures S2C).

Although G0 arrest was observed after both LPS and exogenous IFNβ addition, based on expression levels of cell-cycle-associated transcripts and protein expression of cell-cycle marker MCM2,

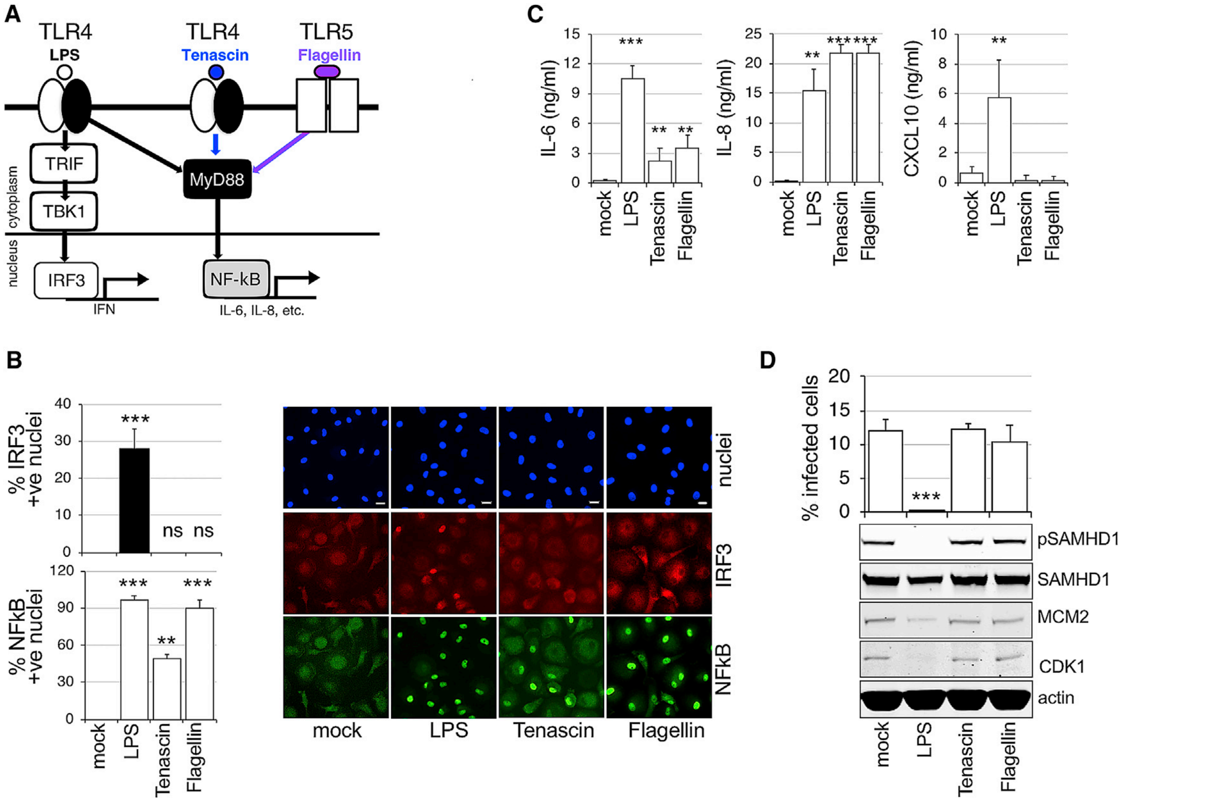

Figure 2. TLR4-Mediated Activation of SAMHD1 Is MyD88 Independent

(A) Diagram of TLR4 activation. LPS activates both MyD88-dependent and -independent signaling pathways. Tenascin-C (TNC) and Flagellin (FLA) activate only the MyD88-dependent pathway leading to NF-κB translocation into the nucleus.

(B) IRF3/NF-κB translocation assay. Cells were exposed to TNC, FLA, and LPS and 2 h later stained for IRF3/NF-κB. The percentage of cells with nuclear staining was determined (n = 3, mean ± SEM). Scale bars, 20 μm.

(C) MDMs were treated with LPS, TNC, and FLA, and cytokines were measured by ELISA in culture media 24 h later.

(D) MDMs treated with TNC, FLA, and LPS were infected by VSV-G-pseudotyped HIV-1 18 h later. The percentage of infected cells was determined 48 h post-infection (n = 3, mean ± SEM). Cells from a representative donor were used for immunoblotting.

***$p \leq 0.001$; **$p \leq 0.01$; ^{ns}p, non-significant, paired *t*-test.

RUXO was unable to rescue this arrest caused by LPS/TLR4 activation but completely rescued G0 arrest after addition of exogenous IFNβ (Figures S2C–S2E). This confirms redundancy of pathways activated by TLR4 that lead to G0 arrest. In concordance with these results, RUXO failed to restore HIV-1 infection from the effect of LPS but completely rescued HIV-1 infection after exposure to exogenous IFNβ (Figure S2E). These data were confirmed by using an IFN receptor antibody instead of RUXO (Figure S2F). As a control, the TLR4 inhibitor TAK242 prevented G0 arrest and restored HIV-1 infection after LPS treatment. As expected, TAK242 could not rescue either after IFNβ treatment. We conclude the existence of two independent pathways that are responsible for G0 arrest in MDMs, both of which are able to potently block HIV-1 infection. The first and early block of HIV-1 infection is via G0 arrest, whereas IFN production represents a second wave.

SAMHD1 Is Directly Responsible for the IFN-Independent HIV-1 Blockade following TLR4 Activation

We have shown previously that the restriction of HIV-1 infection in G0 MDMs can be completely lifted by SAMHD1 depletion (Mlcochova et al., 2017). In the present study, the experimental system involves use of MDMs predominantly in G1-like state, where SAMHD1 is deactivated/phosphorylated at T592. To confirm that LPS-mediated SAMHD1 activation/dephosphorylation is responsible for block to HIV-1 infection during IFN-independent G0 arrest, we employed SAMHD1 KD in the presence of RUXO to inhibit the effects of any secreted IFN (Figures 3D and 3E). We knocked down SAMHD1 expression in human MDMs using small interfering RNA (siRNA) and infected MDMs in the presence or absence of LPS in four different donors (Figures 3D and 3E). SAMHD1 KD lifted HIV-1 block in the presence of LPS. Immunoblot confirmed 80% SAMHD1 KD with no effect on the cell-cycle marker MCM2 (Figure 3E).

In order to confirm the role of SAMHD1, we employed Vpx, an accessory protein encoded by lentiviruses such as HIV-2 and simian immunodeficiency virus (SIV), but not HIV-1 (Hrecka et al., 2011; Laguette et al., 2011). Vpx degrades SAMHD1 in a DCAF-dependent fashion (Hrecka et al., 2011). We exposed MDMs to LPS and co-infected cells 18 h later with HIV-1 and SIVmac virus-like particles (SIV VLPs) bearing Vpx (wild-type

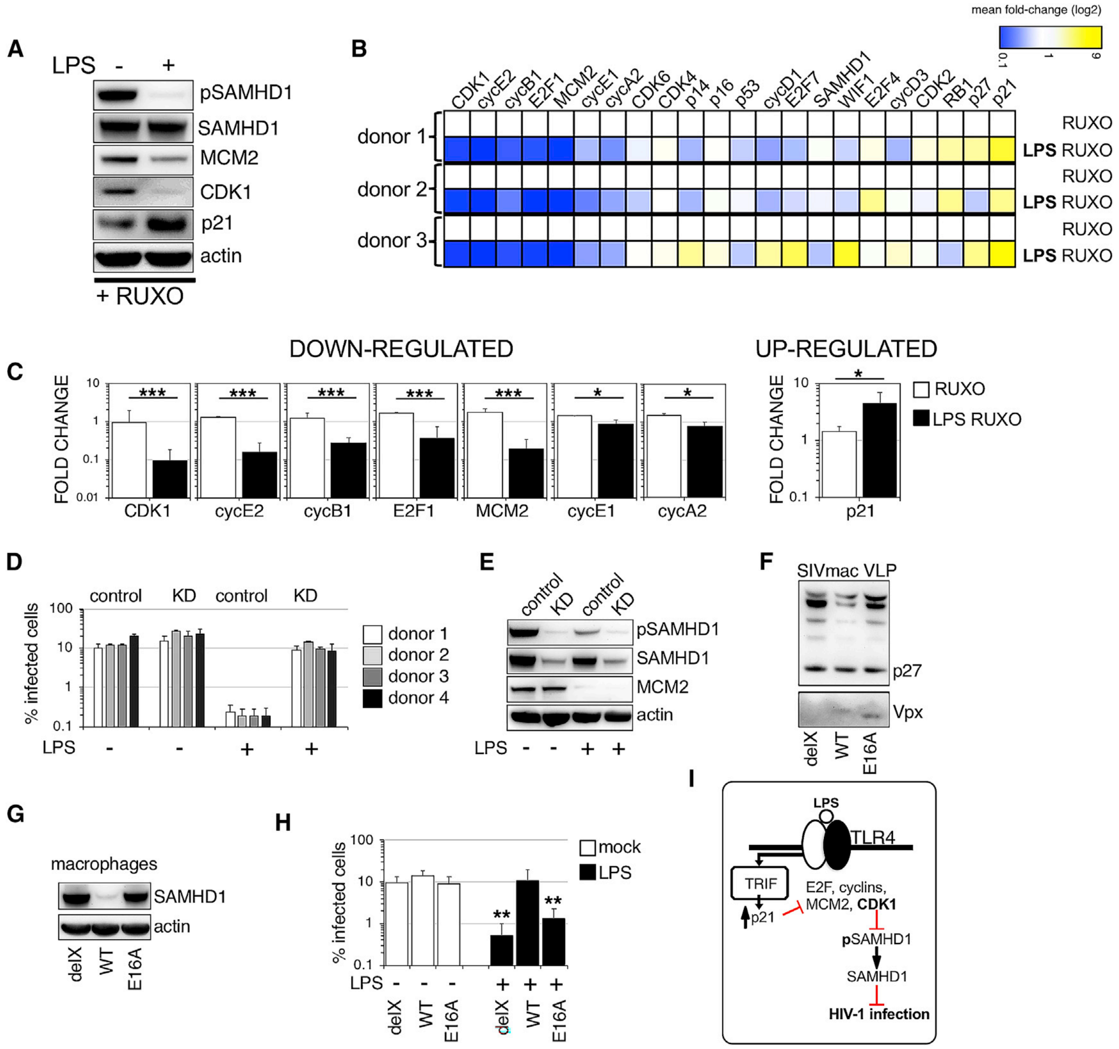

Figure 3. Cell-Cycle Profiling of MDMs following TLR4 Activation and Demonstration that SAMHD1 Mediates the Interferon-Independent Blockade of HIV Infection

(A) MDMs were treated with RUXO 6 h before addition of LPS. Cells from a representative donor were used for immunoblotting 18 h later to detect changes in cell-cycle-associated proteins.

(B) A heatmap depicts differential gene expression patterns of cell-cycle-associated transcripts in MDMs treated with LPS in the presence of RUXO in three donors. The color scale bar corresponds to log-fold expression.

(C) Relative expression levels (fold changes) of statistically significantly changed cell-cycle-associated transcripts after LPS in the presence of RUXO (n = 4 donors, mean ± SEM).

(D and E) MDMs were transfected with control or pool of SAMHD1 siRNAs (KD) and 3 days later treated with RUXO and followed 6 h after that with LPS. Cells were infected in the presence of LPS with VSV-G-pseudotyped HIV-1 GFP 18 h later. The percentage of infected cells was quantified 48 h post-infection. SAMHD1 KD in four different donors. Error bars represent technical triplicates (D). Cells from a representative donor were used for immunoblotting (E).

(F) Immunoblot of SIV virus-like particles (VLPs). delX, SIV VLP with deleted Vpx; E16A, SIV VLP containing mutated Vpx (E16A mutant Vpx does not bind SAMHD1); WT, SIV VLP containing wild-type Vpx.

(G) SAMHD1 degradation in MDMs by SIV VLPs. Equal quantities of SIV VLPs were added to macrophages. Cells from a representative donor were used for immunoblotting.

(H) MDMs were treated ± LPS and infected by VSV-G-pseudotyped HIV-1 in the presence of different SIV VLPs (as indicated). The percentage of infected cells was determined 48 h post-infection (n = 4 donors, mean ± SEM).

(I) Diagram of G0 arrest following TLR4/TRIF activation resulting in block to HIV-1 infection.

***p $\leq$ 0.001; **p $\leq$ 0.01; *p $\leq$ 0.1; ^{ns}p, non-significant, paired t test.

[WT], degrades SAMHD1), no Vpx present (delX, no SAMHD1 degradation), and E16A Vpx mutant that fails to interact with SAMHD1 (Ahn et al., 2012) (E16A, no SAMHD1 degradation) (Figures 3F and 3G). When MDMs were exposed to LPS and infected with HIV-1, only cells where SAMHD1 was exogenously depleted by co-infection with SIV VLP WT were fully susceptible

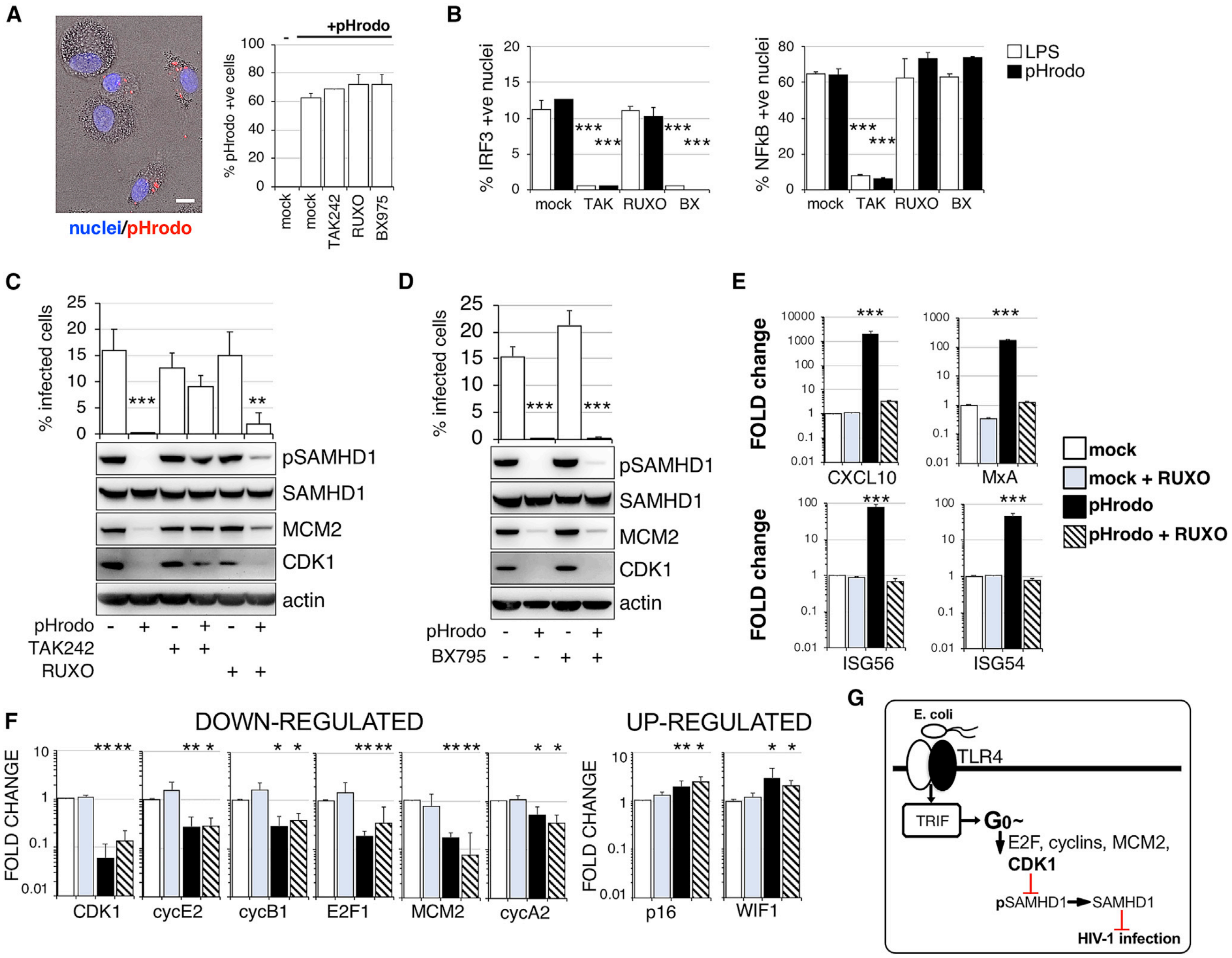

Figure 4. TLR4 Activation by Whole *E. coli* Induces Interferon-Independent G0 Arrest

(A) pHrodo-labeled *E. coli* were added to MDMs for 1 h. MDMs were washed and fixed. 10^4 cells were recorded and analyzed. Percentage of *E. coli*-positive cells was determined using automated cell imaging system Hermes WiScan and ImageJ.

(B) IRF3/NF-κB translocation assay. Cells were exposed to pHrodo *E. coli* in the presence or absence of inhibitors and 2 h later stained for IRF3/NF-κB. The percentage of cells with nuclear staining was determined (n = 3, mean ± SEM). No IRF3 or NF-κB translocation was detected in un-treated cells.

(C and D) MDMs were treated with (C) TAK242 or RUXO 6 h or (D) BX795 2 h before addition of pHrodo *E. coli*. Cells were infected by VSV-G-pseudotyped HIV-1 18 h later. The percentage of infected cells was determined 48 h post-infection (n = 3 donors, mean ± SEM). Cells from a representative donor were used for immunoblotting.

(E and F) Relative expression levels (fold changes) of ISGs (E) and cell-cycle-associated transcripts (F). MDMs were treated with RUXO 6 h before addition of pHrodo *E. coli*. Cells were collected 24 h later (n = 3 donors, mean ± SEM).

(G) Diagram of G0 arrest following exposure to *E. coli* and TLR4/TRIF activation resulting in block to HIV-1 infection.

$^{***}p \leq 0.001$; $^{**}p \leq 0.01$; $^{*}p \leq 0.1$, paired t test.

to infection (Figure 3H). These data highlight the key role that SAMHD1 plays in the TLR4-mediated IFN-independent antiretroviral state in human macrophages (Figure 3I).

Whole Gram-Negative Bacteria Induce IFN-Independent G0 Arrest in Human MDMs

We employed pHrodo *E. coli* BioParticles to activate TLR4 by whole *E. coli* bacteria. BioParticles were incubated with MDMs in the presence or absence of different inhibitors for 1 h at 37°C (Figure 4A). Unbound pHrodo was washed off, and macrophages were incubated overnight. Cell supernatants were then collected for cytokine detection (Figure S4A), and MDMs were infected with HIV-1. First, phagocytosis of pHrodo was unaffected by the presence of TLR4, JAK1/2, or TBK1 inhibitors (Figure 4A). Second, binding/ingestion of pHrodo triggered expression of tumor necrosis factor alpha (TNF-α), IL-6, and IL-8 that was abrogated after TLR4 inhibition, but not by inhibition of the IFN signaling pathway (Figure S4A). This was confirmed by IRF3 and NF-κB translocation assays (Figure 4B). These data show that pHrodo triggers a robust immune response in

MDMs that can be prevented by TLR4 inhibition. Treatment of MDMs with pHrodo induced potent HIV-1 inhibition that was accompanied by G0 arrest and SAMHD1 activation/dephosphorylation at T592 (Figures 4C and 4D). Importantly, TLR4 blockade was able to prevent G0 arrest, SAMHD1 dephosphorylation, and HIV-1 blockade, but neither RUXO nor TBK1 inhibitor BX795 could achieve this, phenocopying experiments with LPS alone (Figure 1). However, when we measured cell-cycle-associated transcripts there were several differences between LPS- and *E. coli*-mediated TLR4 activation, mostly connected to negative regulators of cell cycle. p16 and WIF1 were significantly increased, but no change was detected for p21 (Figures 4E, 4F, and S4B). These data show that gram-negative bacteria can induce IFN-independent G0 arrest and SAMHD1 dephosphorylation/activation in human macrophages (Figure 4G).

DISCUSSION

Here we have shown that macrophages respond to gram-negative bacteria not only by activating the canonical TLR4 pathways involving NF-κB and IRF3, but also by a pathway independent of TBK1 and IRF3, culminating in p21 upregulation and G0 arrest. This previously unrecognized pathway is not dependent or sensitive to blockade of the type I IFN/JAK-STAT signaling axis. Activation of the pathway leads to dephosphorylation of SAMHD1 at a T592 and a specific block to HIV-1 infection that is significantly counteracted by SAMHD1 depletion. Importantly, we have also shown that TLR4 activation by whole *E. coli* bacteria also leads to a similar IFN-independent G0 arrest in human macrophages.

The effect of LPS on cell cycle has been reported in mouse primary cells and murine cell lines (Vairo et al., 1992; Zhang et al., 2017) or in the human cell line, THP-1/U937 (Mytych et al., 2017; Thongngarm et al., 2003). Despite these reports, surprisingly little is known about the mechanism how LPS causes cell-cycle arrest. It has been reported that LPS treatment leads to p21 pathway activation and cell-cycle arrest in monocytic THP-1 (Mytych et al., 2017). In addition, LPS exposure has been linked to ROS production, subsequent DNA damage, and upregulation of p21 expression in monocytes and fibroblasts (Cheng et al., 2015; Mytych et al., 2017). Despite these reports, our data show that neither ROS nor DNA damage seem to be associated with G0 arrest in human MDMs (Figure S3). Another possibility is that strong TLR4 activation triggers an apoptotic program in the cells, and cell-cycle arrest is the first step to apoptosis and cell death (Evan and Vousden, 2001). However, our data do not support this hypothesis because no significant reduction in cell numbers was observed up to 5 days post-LPS treatment (Figure S3G), suggesting survival of activated macrophages. Furthermore, we observed increased levels of p21 after LPS treatment, and p21 has been reported to play an antiapoptotic role (Benson et al., 2009; Gartel and Tyner, 2002; Merched and Chan, 2004). Increased expression of p21 might thus lead to G0 arrest but at the same time prevent apoptosis.

Why would macrophages regulate their cell cycle in response to bacteria? Macrophages are secretory cells vital to the regulation of immune responses and development of inflammation. Even though our previous work showed that MDMs enter G1 without measurable cell division (Mlcochova et al., 2017), many tissue resident macrophages can proliferate (Gomez Perdiguero et al., 2013). One can imagine that cell division of macrophages would benefit the host by increasing the number of effector cells at the center of infection. However, the division of infected cells harboring live pathogen could also lead to doubling of infected cells, an event that can potentially harm the host. Therefore, we speculate that this early cell arrest could be a mechanism for limiting local invasion of gram-negative bacteria into macrophages.

It is also possible that cell-cycle changes are necessary for activation of alternative functions of cell-cycle-associated proteins. G0 or cell-cycle arrested cells will increase expression of p14, p16, p21, or p27 proteins, for example. It has been shown that p21 can suppress IL-1β (Scatizzi et al., 2009), or that p16 inhibits macrophage activity by degradation of IL-1 receptor and thus impairs IL-6 production (Murakami et al., 2012). In addition, previous studies have shown that deficiency in p21 renders mice more susceptible to septic shock (Scatizzi et al., 2009; Trakala et al., 2009); therefore, cell arrest may be a mechanism for limiting production of potentially harmful cytokines.

The relevance of macrophage G0 arrest by LPS may be relevant in HIV pathogenesis, where macrophages will be exposed to gut-derived LPS during inflammation in the acute or chronic phase of HIV. It has been shown that circulating LPS is increased in chronically HIV-1-infected individuals and SIV-infected nonhuman primates (Brenchley et al., 2006). Exploring LPS-mediated regulation in primary human macrophages is therefore important for our understanding of HIV-1 replication and cellular reservoirs (Watters et al., 2013).

Our study is based on primary MDMs rather than tissue-derived macrophages, such as those in lymph nodes, gut, or central nervous system. We utilized a differentiation protocol that led to high proportions of macrophages in G1 in order to be able to clearly study the effect of LPS on cell cycle. Although we previously reported 10%–20% of *ex vivo* peritoneal mouse macrophages and microglial cells to be in G1 (Mlcochova et al., 2017), it is difficult to know the proportions of macrophages in G1 across diverse human tissues. Even if 10%–20% of macrophages *in vivo* are in G1, this is nonetheless significant, and LPS responses in these cells warrant specific characterization.

In summary, our data show that TLR4 activation by LPS or whole bacteria regulates the cell cycle in human primary macrophages through a non-canonical mechanism that is TRIF dependent. This culminates in G0 arrest and activation of the antiviral protein SAMHD1, suggesting that macrophages can rapidly achieve a heightened state of alert in response to gram-negative bacteria prior to type I IFN secretion. Finally, given that macrophage G0 arrest occurs following DNA damage (Mlcochova et al., 2018), histone deacetylase (HDAC) inhibition (Mlcochova et al., 2017), and immune stimuli, we conclude that cell-cycle regulation appears to be a conserved and important response to danger signals in human macrophages.

STAR★METHODS

Detailed methods are provided in the online version of this paper and include the following:

- KEY RESOURCES TABLE
- LEAD CONTACT AND MATERIALS AVAILABILITY

- EXPERIMENTAL MODEL AND SUBJECT DETAILS
  - Cell lines and viruses
  - Monocyte isolation and differentiation
  - Ethics Statement
- METHOD DETAILS
  - Infection of primary cells using full-length and VSV-G pseudotyped HIV-1 viruses
  - SDS-PAGE and Immunoblots
  - SAMHD1 knock-down by siRNA
  - Quantitative PCR
  - Immunofluorescence
  - Phagocytosis assay using pHrodo Bioparticles
  - ELISA
- QUANTIFICATION AND STATISTICAL ANALYSIS
- DATA AND CODE AVAILABILITY

SUPPLEMENTAL INFORMATION

Supplemental Information can be found online at https://doi.org/10.1016/j.celrep.2020.03.008.

ACKNOWLEDGMENTS

This work was funded by a Wellcome Trust Senior Fellowship in Clinical Science to R.K.G. (WT108082AIA) and the University of Cambridge National Institute for Health Research Biomedical Research Centre.

AUTHOR CONTRIBUTIONS

P.M., R.K.G., and L.Z.-A. designed experiments; P.M. and R.K.G. wrote the manuscript; P.M. and H.W. performed experiments; P.M., R.K.G., and L.Z.-A. analyzed data.

DECLARATION OF INTERESTS

The authors declare no competing interests.

Received: November 4, 2019
Revised: January 9, 2020
Accepted: February 28, 2020
Published: March 24, 2020

REFERENCES

Ahn, J., Hao, C., Yan, J., DeLucia, M., Mehrens, J., Wang, C., Gronenborn, A.M., and Skowronski, J. (2012). HIV/simian immunodeficiency virus (SIV) accessory virulence factor Vpx loads the host cell restriction factor SAMHD1 onto the E3 ubiquitin ligase complex CRL4DCAF1. J. Biol. Chem. *287*, 12550–12558.

Akira, S., and Takeda, K. (2004). Toll-like receptor signalling. Nat. Rev. Immunol. *4*, 499–511.

Arnold, L.H., Groom, H.C.T., Kunzelmann, S., Schwefel, D., Caswell, S.J., Ordonez, P., Mann, M.C., Rueschenbaum, S., Goldstone, D.C., Pennell, S., et al. (2015). Phospho-dependent Regulation of SAMHD1 Oligomerisation Couples Catalysis and Restriction. PLoS Pathog. *11*, e1005194.

Benson, E.K., Zhao, B., Sassoon, D.A., Lee, S.W., and Aaronson, S.A. (2009). Effects of p21 deletion in mouse models of premature aging. Cell Cycle *8*, 2002–2004.

Bernstein, M.S., Tong-Starksen, S.E., and Locksley, R.M. (1991). Activation of human monocyte-derived macrophages with lipopolysaccharide decreases human immunodeficiency virus replication in vitro at the level of gene expression. J. Clin. Invest. *88*, 540–545.

Besnier, C., Takeuchi, Y., and Towers, G. (2002). Restriction of lentivirus in monkeys. Proc. Natl. Acad. Sci. USA *99*, 11920–11925.

Bhattacharya, A., Wang, Z., White, T., Buffone, C., Nguyen, L.A., Shepard, C.N., Kim, B., Demeler, B., Diaz-Griffero, F., and Ivanov, D.N. (2016). Effects of T592 phosphomimetic mutations on tetramer stability and dNTPase activity of SAMHD1 can not explain the retroviral restriction defect. Sci. Rep. *6*, 31353.

Brenchley, J.M., Price, D.A., Schacker, T.W., Asher, T.E., Silvestri, G., Rao, S., Kazzaz, Z., Bornstein, E., Lambotte, O., Altmann, D., et al. (2006). Microbial translocation is a cause of systemic immune activation in chronic HIV infection. Nat. Med. *12*, 1365–1371.

Cheng, R., Choudhury, D., Liu, C., Billet, S., Hu, T., and Bhowmick, N.A. (2015). Gingival fibroblasts resist apoptosis in response to oxidative stress in a model of periodontal diseases. Cell Death Discov. *1*, 15046.

Cribier, A., Descours, B., Valadão, A.L.C., Laguette, N., and Benkirane, M. (2013). Phosphorylation of SAMHD1 by cyclin A2/CDK1 regulates its restriction activity toward HIV-1. Cell Rep. *3*, 1036–1043.

Dey, A., She, H., Kim, L., Boruch, A., Guris, D.L., Carlberg, K., Sebti, S.M., Woodley, D.T., Imamoto, A., and Li, W. (2000). Colony-stimulating factor-1 receptor utilizes multiple signaling pathways to induce cyclin D2 expression. Mol. Biol. Cell *11*, 3835–3848.

Evan, G.I., and Vousden, K.H. (2001). Proliferation, cell cycle and apoptosis in cancer. Nature *411*, 342–348.

Franchin, G., Zybarth, G., Dai, W.W., Dubrovsky, L., Reiling, N., Schmidtmayerova, H., Bukrinsky, M., and Sherry, B. (2000). Lipopolysaccharide inhibits HIV-1 infection of monocyte-derived macrophages through direct and sustained down-regulation of CC chemokine receptor 5. J. Immunol. *164*, 2592–2601.

Gartel, A.L., and Tyner, A.L. (2002). The role of the cyclin-dependent kinase inhibitor p21 in apoptosis. Mol. Cancer Ther. *1*, 639–649.

Geonnotti, A.R., Bilska, M., Yuan, X., Ochsenbauer, C., Edmonds, T.G., Kappes, J.C., Liao, H.-X., Haynes, B.F., and Montefiori, D.C. (2010). Differential inhibition of human immunodeficiency virus type 1 in peripheral blood mononuclear cells and TZM-bl cells by endotoxin-mediated chemokine and gamma interferon production. AIDS Res. Hum. Retroviruses *26*, 279–291.

Goldstone, D.C., Ennis-Adeniran, V., Hedden, J.J., Groom, H.C.T., Rice, G.I., Christodoulou, E., Walker, P.A., Kelly, G., Haire, L.F., Yap, M.W., et al. (2011). HIV-1 restriction factor SAMHD1 is a deoxynucleoside triphosphate triphosphohydrolase. Nature *480*, 379–382.

Gomez Perdiguero, E., Schulz, C., and Geissmann, F. (2013). Development and homeostasis of "resident" myeloid cells: the case of the microglia. Glia *61*, 112–120.

Goujon, C., Arfi, V., Pertel, T., Luban, J., Lienard, J., Rigal, D., Darlix, J.-L., and Cimarelli, A. (2008). Characterization of simian immunodeficiency virus SIVSM/human immunodeficiency virus type 2 Vpx function in human myeloid cells. J. Virol. *82*, 12335–12345.

Herrmann, A., Wittmann, S., Thomas, D., Shepard, C.N., Kim, B., Ferreirós, N., and Gramberg, T. (2018). The SAMHD1-mediated block of LINE-1 retroelements is regulated by phosphorylation. Mob. DNA *9*, 11.

Hrecka, K., Hao, C., Gierszewska, M., Swanson, S.K., Kesik-Brodacka, M., Srivastava, S., Florens, L., Washburn, M.P., and Skowronski, J. (2011). Vpx relieves inhibition of HIV-1 infection of macrophages mediated by the SAMHD1 protein. Nature *474*, 658–661.

Iwasaki, A., and Medzhitov, R. (2004). Toll-like receptor control of the adaptive immune responses. Nat. Immunol. *5*, 987–995.

Kawai, T., Takeuchi, O., Fujita, T., Inoue, J., Mühlradt, P.F., Sato, S., Hoshino, K., and Akira, S. (2001). Lipopolysaccharide stimulates the MyD88-independent pathway and results in activation of IFN-regulatory factor 3 and the expression of a subset of lipopolysaccharide-inducible genes. J. Immunol. *167*, 5887–5894.

Kornbluth, R.S., Oh, P.S., Munis, J.R., Cleveland, P.H., and Richman, D.D. (1989). Interferons and bacterial lipopolysaccharide protect macrophages from productive infection by human immunodeficiency virus in vitro. J. Exp. Med. *169*, 1137–1151.

Laguette, N., Sobhian, B., Casartelli, N., Ringeard, M., Chable-Bessia, C., Ségéral, E., Yatim, A., Emiliani, S., Schwartz, O., and Benkirane, M. (2011). SAMHD1 is the dendritic- and myeloid-cell-specific HIV-1 restriction factor counteracted by Vpx. Nature *474*, 654–657.

Lahouassa, H., Daddacha, W., Hofmann, H., Ayinde, D., Logue, E.C., Dragin, L., Bloch, N., Maudet, C., Bertrand, M., Gramberg, T., et al. (2012). SAMHD1 restricts the replication of human immunodeficiency virus type 1 by depleting the intracellular pool of deoxynucleoside triphosphates. Nat. Immunol. *13*, 223–228.

Merched, A.J., and Chan, L. (2004). Absence of p21Waf1/Cip1/Sdi1 modulates macrophage differentiation and inflammatory response and protects against atherosclerosis. Circulation *110*, 3830–3841.

Midwood, K., Sacre, S., Piccinini, A.M., Inglis, J., Trebaul, A., Chan, E., Drexler, S., Sofat, N., Kashiwagi, M., Orend, G., et al. (2009). Tenascin-C is an endogenous activator of Toll-like receptor 4 that is essential for maintaining inflammation in arthritic joint disease. Nat. Med. *15*, 774–780.

Midwood, K.S., Chiquet, M., Tucker, R.P., and Orend, G. (2016). Tenascin-C at a glance. J. Cell Sci. *129*, 4321–4327.

Mlcochova, P., Sutherland, K.A., Watters, S.A., Bertoli, C., de Bruin, R.A., Rehwinkel, J., Neil, S.J., Lenzi, G.M., Kim, B., Khwaja, A., et al. (2017). A G1-like state allows HIV-1 to bypass SAMHD1 restriction in macrophages. EMBO J. *36*, 604–616.

Mlcochova, P., Caswell, S.J., Taylor, I.A., Towers, G.J., and Gupta, R.K. (2018). DNA damage induced by topoisomerase inhibitors activates SAMHD1 and blocks HIV-1 infection of macrophages. EMBO J. *37*, 50–62.

Munn, D.H., Pressey, J., Beall, A.C., Hudes, R., and Alderson, M.R. (1996). Selective activation-induced apoptosis of peripheral T cells imposed by macrophages. A potential mechanism of antigen-specific peripheral lymphocyte deletion. J. Immunol. *156*, 523–532.

Murakami, Y., Mizoguchi, F., Saito, T., Miyasaka, N., and Kohsaka, H. (2012). p16(INK4a) exerts an anti-inflammatory effect through accelerated IRAK1 degradation in macrophages. J. Immunol. *189*, 5066–5072.

Mytych, J., Romerowicz-Misielak, M., and Koziorowski, M. (2017). Long-term culture with lipopolysaccharide induces dose-dependent cytostatic and cytotoxic effects in THP-1 monocytes. Toxicol. In Vitro *42*, 1–9.

Park, B.S., and Lee, J.O. (2013). Recognition of lipopolysaccharide pattern by TLR4 complexes. Exp. Mol. Med. *45*, e66.

Reinhard, C., Bottinelli, D., Kim, B., and Luban, J. (2014). Vpx rescue of HIV-1 from the antiviral state in mature dendritic cells is independent of the intracellular deoxynucleotide concentration. Retrovirology *11*, 12.

Scatizzi, J.C., Mavers, M., Hutcheson, J., Young, B., Shi, B., Pope, R.M., Ruderman, E.M., Samways, D.S.K., Corbett, J.A., Egan, T.M., and Perlman, H. (2009). The CDK domain of p21 is a suppressor of IL-1β-mediated inflammation in activated macrophages. Eur. J. Immunol. *39*, 820–825.

Schlaepfer, E., Rochat, M.-A., Duo, L., and Speck, R.F. (2014). Triggering TLR2, -3, -4, -5, and -8 reinforces the restrictive nature of M1- and M2-polarized macrophages to HIV. J. Virol. *88*, 9769–9781.

Szaniawski, M.A., Spivak, A.M., Cox, J.E., Catrow, J.L., Hanley, T., Williams, E.S.C.P., Tremblay, M.J., Bosque, A., and Planelles, V. (2018). SAMHD1 Phosphorylation Coordinates the Anti-HIV-1 Response by Diverse Interferons and Tyrosine Kinase Inhibition. MBio *9*, e00819-18.

Thongngarm, T., Jenkins, J.K., Ndebele, K., and McMurray, R.W. (2003). Estrogen and progesterone modulate monocyte cell cycle progression and apoptosis. Am. J. Reprod. Immunol. *49*, 129–138.

Trakala, M., Arias, C.F., García, M.I., Moreno-Ortiz, M.C., Tsilingiri, K., Fernández, P.J., Mellado, M., Díaz-Meco, M.T., Moscat, J., Serrano, M., et al. (2009). Regulation of macrophage activation and septic shock susceptibility via p21(WAF1/CIP1). Eur. J. Immunol. *39*, 810–819.

Tsang, J., Chain, B.M., Miller, R.F., Webb, B.L.J., Barclay, W., Towers, G.J., Katz, D.R., and Noursadeghi, M. (2009). HIV-1 infection of macrophages is dependent on evasion of innate immune cellular activation. AIDS *23*, 2255–2263.

Vairo, G., Royston, A.K., and Hamilton, J.A. (1992). Biochemical events accompanying macrophage activation and the inhibition of colony-stimulating factor-1-induced macrophage proliferation by tumor necrosis factor-α, interferon-γ, and lipopolysaccharide. J. Cell. Physiol. *151*, 630–641.

Valle-Casuso, J.C., Allouch, A., David, A., Lenzi, G.M., Studdard, L., Barré-Sinoussi, F., Müller-Trutwin, M., Kim, B., Pancino, G., and Sáez-Cirión, A. (2017). p21 Restricts HIV-1 in Monocyte-Derived Dendritic Cells through the Reduction of Deoxynucleoside Triphosphate Biosynthesis and Regulation of SAMHD1 Antiviral Activity. J. Virol. *91*, e01324-17.

Verani, A., Scarlatti, G., Comar, M., Tresoldi, E., Polo, S., Giacca, M., Lusso, P., Siccardi, A.G., and Vercelli, D. (1997). C-C chemokines released by lipopolysaccharide (LPS)-stimulated human macrophages suppress HIV-1 infection in both macrophages and T cells. J. Exp. Med. *185*, 805–816.

Verani, A., Sironi, F., Siccardi, A.G., Lusso, P., and Vercelli, D. (2002). Inhibition of CXCR4-tropic HIV-1 infection by lipopolysaccharide: evidence of different mechanisms in macrophages and T lymphocytes. J. Immunol. *168*, 6388–6395.

Wang, H., Sun, J., and Goldstein, H. (2008). Human immunodeficiency virus type 1 infection increases the in vivo capacity of peripheral monocytes to cross the blood-brain barrier into the brain and the in vivo sensitivity of the blood-brain barrier to disruption by lipopolysaccharide. J. Virol. *82*, 7591–7600.

Watters, S.A., Mlcochova, P., and Gupta, R.K. (2013). Macrophages: the neglected barrier to eradication. Curr. Opin. Infect. Dis. *26*, 561–566.

Welbourn, S., and Strebel, K. (2016). Low dNTP levels are necessary but may not be sufficient for lentiviral restriction by SAMHD1. Virology *488*, 271–277.

White, T.E., Brandariz-Nuñez, A., Valle-Casuso, J.C., Amie, S., Nguyen, L.A., Kim, B., Tuzova, M., and Diaz-Griffero, F. (2013). The retroviral restriction ability of SAMHD1, but not its deoxynucleotide triphosphohydrolase activity, is regulated by phosphorylation. Cell Host Microbe *13*, 441–451.

Xaus, J., Cardó, M., Valledor, A.F., Soler, C., Lloberas, J., and Celada, A. (1999). Interferon γ induces the expression of p21waf-1 and arrests macrophage cell cycle, preventing induction of apoptosis. Immunity *11*, 103–113.

Zhang, C., Cui, G., Chen, Y., and Fan, K. (2007). Antitumor effect of interferon-alpha on U937 human acute leukemia cells in vitro and its molecular mechanism. J. Huazhong Univ. Sci. Technolog. Med. Sci. *27*, 513–515.

Zhang, K., Song, F., Lu, X., Chen, W., Huang, C., Li, L., Liang, D., Cao, S., and Dai, H. (2017). MicroRNA-322 inhibits inflammatory cytokine expression and promotes cell proliferation in LPS-stimulated murine macrophages by targeting NF-κB1 (p50). Biosci. Rep. *37*, BSR20160239.

STAR★METHODS

KEY RESOURCES TABLE

REAGENT or RESOURCE	SOURCE	IDENTIFIER
Antibodies		
Rabbit Anti-Human SAMHD1 Polyclonal Antibody	Proteintech	Cat# 12586-1-AP, RRID:AB_2183496
Mouse Anti-Actin, beta Monoclonal Antibody	Abcam	Cat# ab6276, RRID:AB_2223210
Rabbit polyclonal CDK1 Antibody	Bethyl	Cat# A303-664A, RRID:AB_11204758
Mouse Anti-BM28 Monoclonal Antibody	BD Biosciences	Cat# 610701, RRID:AB_398024
Phospho-SAMHD1 (Thr592) (D7O2M) Rabbit mAb antibody	Cell Signaling Technology	Cat# 89930, RRID:AB_2800147
Mouse Anti-P21 Monoclonal antibody, Unconjugated, Clone f-5	Santa Cruz Biotechnology	Cat# sc-6246, RRID:AB_628073
IRF-3 (D6I4C) XP® antibody	Cell Signaling Technology	Cat# 11904, RRID:AB_2722521
NFkappaB p65 (F-6) antibody	Santa Cruz Biotechnology	Cat# sc-8008, RRID:AB_628017
Purified anti-H2A.X Phospho (Ser139) antibody	BioLegend	Cat# 613402, RRID:AB_315795
53BP1 antibody	BD Biosciences	Cat# 612522, RRID:AB_2206766
Anti-IFNα/β Receptor	PBL Interferon Source	N/A
Mouse IgG2A Isotype Control (Clone 20102) antibody	R and D Systems	Cat# MAB003, RRID:AB_357345
Rat Anti-IL-6 Monoclonal Antibody, Unconjugated, Clone MQ2-13A5	BD Biosciences	Cat# 554543, RRID:AB_398568
Mouse Anti-IL-8 Monoclonal Antibody, Unconjugated, Clone G265-5	BD Biosciences	Cat# 554716, RRID:AB_395526
Mouse Anti-TNF Monoclonal Antibody, Unconjugated, Clone MAb1	BD Biosciences	Cat# 551220, RRID:AB_394098
Rabbit Anti-Human TICAM1 Polyclonal Antibody, Unconjugated	GeneTex	Cat# GTX104744, RRID:AB_1241389
TBK1 Antibody	Bethyl	Cat# A300-093A, RRID:AB_2303002
Bacterial and Virus Strains		
VSV-G HIV-1 GFP virus	Besnier et al., 2002	N/A
SIVmac Virus like particles	Goujon et.al., 2008; Reinhard et al., 2014	N/A
Biological Samples		
PBMC from HIV seronegative donors	This study	N/A
Chemicals, Peptides, and Recombinant Proteins		
AB Human Serum	Sigma	#H4522
FBS	Biosera	N/A
BX795	APEXBIO	#A8222
Ruxolitinib	Cell guidance system	#SM87
Recombinant human IFN-b	Peprotech	#300-02BC
Recombinant human Tenascin C	R and D	#3358-TC-050
Polymyxin B	Sigma	#P4932
LPS	InvivoGen	#tlrl-prslps
TAK242	Milllipore	#614316
Recombinant Flagellin protein	Abcam	#Ab201366
DMEM, high glucose, pyruvate	Invitrogen	#41966052
RPMI 1640 medium	Invitrogen	#21875091

(*Continued on next page*)

Continued

REAGENT or RESOURCE	SOURCE	IDENTIFIER
Opti-MEM	Invitrogen	#31985047
Fugene HD transfection reagent	Promega	#E2311
DAPI	Sigma	#10236276001
PhosSTOP	Sigma	#4906845001
Fast SYRB green master mix	ThermoFisher	#4385610
Total RNA Purification Kit	Norgen	#17200
Critical Commercial Assays		
CellRox	Molecular Probes	#C10444
NuPAGE 4-12% Bis-Tris Protein Gel	Invitrogen	#NP0322BOX
pHrodo E.coli BioParticles	ThermoFisher	#P35361
SuperScript III First-Strand Synthesis System	ThermoFisher	#18080051
Amersham ECL Prime Western Blotting Detection Reagent	GE Healthcare	#RPN2232
Experimental Models: Cell Lines		
293T	Laboratory of G. Towers (UCL)	N/A
Oligonucleotides		
CDK1 FWD 5′TGAGGAACGGGGTCCTCTAA 3′	Invitrogen	N/A
CDK1 R 5′ A TGGCT ACCACTTGA CCTGT 3′	Invitrogen	N/A
CDK2 F 5′ AAGTTGACGGGAGAG GTGGT 3′	Invitrogen	N/A
CDK2 R 5′ TGATGAGGGGAAGAG GAATG 3′	Invitrogen	N/A
CDK4 F 5′ CAGATGGCACTTACACCCGT 3′	Invitrogen	N/A
CDK4 R 5′ CAGCCCAATCAGGTC AAAGA 3′	Invitrogen	N/A
CDK6 F 5′ CGTGGTCAGGTTGTT TGATGT 3′	Invitrogen	N/A
CDK6 R 5′ CGGTGTGAATGAAGAA AGTCC 3′	Invitrogen	N/A
Cyclin A2 F 5′ AAGACGAGACGGGTTGC 3′	Invitrogen	N/A
Cyclin A2 R 5′ GGCTGTTTACTGTTTGCTTTCC 3′	Invitrogen	N/A
Cyclin B1 F 5′ TTCTGGATAATGGTGAATGGAC 3′	Invitrogen	N/A
Cyclin B1 R 5′ ATGTGGCATACTTGTTCTTGAC 3′	Invitrogen	N/A
Cyclin D1 F 5′ AGATGAAGGAGACCATCCCCC 3′	Invitrogen	N/A
Cyclin D1 R 5′ CCACTTGAGCTTGTTCACCA 3′	Invitrogen	N/A
Cyclin D3 F 5′ GGCCGGGGACCGAAACT 3′	Invitrogen	N/A
Cyclin D3 R 5′CAGTGGCGAAGTGTTTACAAAGT 3′	Invitrogen	N/A
Cyclin E1 F 5′ CCGGTATATGGCGACACAAG 3′	Invitrogen	N/A
Cyclin E1 R 5′ TACGCAAACTGGTGCAACTT 3′	Invitrogen	N/A

(*Continued on next page*)

Continued

REAGENT or RESOURCE	SOURCE	IDENTIFIER
Cyclin E2 F 5′ TCTCCTGGCTAAATCTCTTTCTCC 3′	Invitrogen	N/A
Cyclin E2 R 5′ ACTGTCCCACTCCAAACCTG 3′	Invitrogen	N/A
E2F1 F 5′ TGCCAAGAAGTCCAAGA ACCA 3′	Invitrogen	N/A
E2F1 R 5′ GTCAACCCCTCAAGCCGTC 3′	Invitrogen	N/A
E2F4 F 5′ CGGACCCAACCCTTCT ACCT 3′	Invitrogen	N/A
E2F4 R 5′ GGGGCAAACACTTCTGAGGA 3′	Invitrogen	N/A
E2F7 F 5′ CCTTTAGCCCACCCAGTATTT 3′	Invitrogen	N/A
E2F7 R 5′ A TCCCTCTCTGACCCTGACC 3′	Invitrogen	N/A
MCM2 F 5′CACCCGAAGCTCAACCA GAT 3′	Invitrogen	N/A
MCM2 R 5′ATCATGGACTCGATGTG CCG 3′	Invitrogen	N/A
RB1 F 5′AAAGGACCGAGAAGGACCA 3′	Invitrogen	N/A
RB1 R 5′AAGGCTGAGGTTGCTTGTGT 3′	Invitrogen	N/A
WIF1 F 5′TCTGTTCAAAGCCTGTCTGC 3′	Invitrogen	N/A
WIF1 R 5′ACATTGGCATTTGTTGGGTT 3′	Invitrogen	N/A
p14 F 5′GAGTGAGGGTTTTCGTGGTTC 3′	Invitrogen	N/A
p14 R 5′ACGGGTCGGGTGAGAGTG 3′	Invitrogen	N/A
p16 F 5′ CGGCTGACTGGCTGGC 3′	Invitrogen	N/A
p16 R 5′ GGGTCGGGTGAGAGTGG3′	Invitrogen	N/A
p21 F 5′GCCGAAGTCAGTTCCTTGTG 3′	Invitrogen	N/A
p21 R 5′TCGAAGTTCCATCGCTCACG 3′	Invitrogen	N/A
p27 F 5′A TGTTTCAGACGGTTCCCCA 3′	Invitrogen	N/A
p27 R 5′TCCAACGCTTTTAGAGGCAG 3′	Invitrogen	N/A
p53 F 5′AAGTCTAGAGCCACCGTCCA 3′	Invitrogen	N/A
p53 R 5′TTTCAGGAAGTAGTTTCCATAGGT 3′	Invitrogen	N/A
CXCL10 F 5′ TGGCATTCAAGGAGTACC TC 3′	Sigma	N/A
CXCL10 R 5′ TTGTAGCAATGATCTCAACACG 3′	Sigma	N/A
ISG56 F 5′CCT CCT TGG GTT CGT CTA CA 3′	Sigma	N/A
ISG56 R 5′GGC TGA TAT CTG GGT GCC TA 3′	Sigma	N/A
ISG54 F 5′CAGCTGAGAATTGCACTGC AA 3′	Sigma	N/A
ISG54 R 5′CGT AGGCTGCTCTCCAAG GA 3′	Sigma	N/A
MxA F 5′ATC CTG GGA TTT TGG GGC TT 3′	Sigma	N/A
MxA R 5′CCG CTT GTC GCT GGT GTC G 3′	Sigma	N/A
SAMHD1 F 5′TTGTGCTAGAGATAAGGAAGTTGG 3′	Invitrogen	N/A
SAMHD1 R 5′TGTGTTGATAAGCTCTACGGTG 3′	Invitrogen	N/A
GAPDH F 5′ACC CAG AAG ACT GTG GAT GG 3′	Sigma	N/A

(*Continued on next page*)

Continued		
REAGENT or RESOURCE	SOURCE	IDENTIFIER
GAPDH R 5′TTC TAG ACG GCA GGT CAG GT 3′	Sigma	N/A
ON-TARGETplus Human SAMHD1 siRNA	Dharmacon	#L-013950-01
Control siRNA	Santa Cruz	#sc-37007
TBK1 siRNA	Santa Cruz	#sc-39058
TICAM1 siRNA	OriGene	#SR315629
Recombinant DNA		
GFP-encoding genome CSGW	Laboratory of G. Towers	N/A
p8.91	Laboratory of G. Towers	N/A
pMDG	Laboratory of G. Towers	N/A
SIVmac packaging plasmid encoding accessory genes	Laboratory of G. Towers	N/A
pCDNA Vpx E16A	Laboratory of J. Luban, Reinhard et al., 2014	N/A
Software and Algorithms		
ImageJ		https://imagej.nih.gov/ij/
Other		
Amersham Hybond P 0.45 PVDF blotting membrane	GE Healthcare	#10600023

LEAD CONTACT AND MATERIALS AVAILABILITY

Further information and requests for resources and reagents should be directed to and will be fulfilled by the Lead Contact, Prof. Ravi Gupta (rkg20@cam.ac.uk).

This study did not generate unique new reagents.

EXPERIMENTAL MODEL AND SUBJECT DETAILS

Cell lines and viruses

293T cells were cultured in DMEM complete (DMEM supplemented with 100 U/ml penicillin, 0.1 mg/ml streptomycin, and 10% FCS). VSV-G HIV-1 GFP virus was produced by transfection of 293T with GFP-encoding genome CSGW, packaging plasmid p8.91 and pMDG as previously described (Besnier et al., 2002). SIVmac Virus like particles (VLP) containing Vpx were prepared as previously described (Goujon et al., 2008; Reinhard et al., 2014).

Monocyte isolation and differentiation

PBMC were prepared from HIV seronegative male and female donors (after informed consent was obtained), by density-gradient centrifugation (Lymphoprep, Axis-Shield, UK). Monocyte-derived macrophages (MDM) were prepared by adherence with washing of non-adherent cells after 2h, with subsequent maintenance of adherent cells in RPMI 1640 medium supplemented with 10% human serum and MCSF (10ng/ml) for 3 days and then differentiated for a further 4 days in RPMI 1640 medium supplemented with 10% fetal calf sera without M-CSF.

Ethics Statement

Adult subjects provided written informed consent. Primary Macrophage & Dendritic Cell Cultures from Healthy Volunteer Blood Donors has been reviewed and granted ethical permission by the National Research Ethics Service through The Joint UCL/UCLH Committees on the Ethics of Human Research (Committee Alpha) 2nd of December 2009. Reference number 06/Q0502/92.

METHOD DETAILS

Infection of primary cells using full-length and VSV-G pseudotyped HIV-1 viruses

GFP containing VSV-G pseudotyped HIV-1 was added to MDM and after 4h incubation removed and cells were washed in culture medium. The percentage of infected cells was determined 48h post-infection by Hermes WiScan automated cell-imaging system (IDEA Bio-Medical Ltd. Rehovot, Israel) and analyzed using MetaMorph and ImageJ software. In the experiments when LPS was used cells were stimulated with 10ng/ml of LPS 18h before infection unless stated otherwise. 10^4 cells were recorded and analyzed.

SDS-PAGE and Immunoblots

Cells were lysed in reducing Laemmli SDS sample buffer containing PhosSTOP (Phosphatase Inhibitor Cocktail Tablets, Roche, Switzerland) at 96°C for 10 minutes and the proteins separated on NuPAGE® Novex® 4%–12% Bis-Tris Gels. Subsequently, the proteins were transferred onto PVDF membranes (Millipore, Billerica, MA, USA), the membranes were quenched, and proteins detected using specific antibodies. Labeled protein bands were detected using Amersham ECL Prime Western Blotting Detection Reagent (GE Healthcare, USA) and Amersham Hyperfilm or AlphaInnotech CCD camera. Protein band intensities were recorded and quantified using AlphaInnotech CCD camera and AlphaView software (ProteinSimple, San Jose, California, USA).

SAMHD1 knock-down by siRNA

1x10e5 MDM differentiated in MCSF for 4 days were transfected with 20pmol of siRNA (L-013950-01, Dharmacon) using *Lipofectamine RNAiMAX* Transfection Reagent (Invitrogen). Transfection medium was replaced after 18h with RPMI 1640 medium supplemented with 10% FCS and cells cultured for additional 3 days before infection.

Quantitative PCR

Total RNA was isolated from macrophages using the Total RNA Purification Kit from Norgen Biotek (Thorold, Canada). cDNA was synthesized using Superscript III Reverse Transcriptase (Thermo Fisher Scientific) using 500ng of template RNA. qPCR was performed on ABI 7300 machine (Thermo Fisher Scientific) using Fast SYRB green master mix (Thermo Fisher Scientific). Expression levels of target genes were normalized to glyceraldehyde-3-phosphate dehydrogenase (GAPDH) as previously described (Tsang et al., 2009). See primer sequences in STAR Methods.

Immunofluorescence

MDMs were fixed in 3% PFA, quenched with 50 mM NH_4Cl and permeabilized with 0.1% Triton X-100 in PBS or 90% Methanol. After blocking in PBS/1% FCS, MDMs were labeled for 1 hour with primary antibodies diluted in PBS/1% FCS, washed and labeled again with Alexa Fluor secondary antibodies for 1 hour. Cells were washed in PBS/1% FCS and stained with DAPI in PBS for 20 minutes. Labeled cells were detected using Hermes WiScan automated cell-imaging system (IDEA Bio-Medical Ltd. Rehovot, Israel) and analyzed using MetaMorph and ImageJ software. On average 10^4 cells were recorded and analyzed in each experiment.

Phagocytosis assay using pHrodo Bioparticles

MDM were exposed to 0.25ug pHrodo (a pH-sensitive, rhodamine-based dye)-labeled *E. coli* for 1h. MDM were washed 3x in PBS and fixed. The percentage of *E.coli* positive cells was determined using Hermes WiScan automated cell-imaging system (IDEA Bio-Medical Ltd. Rehovot, Israel) and analyzed using MetaMorph and ImageJ software. 10^4 cells were recorded and analyzed.

ELISA

Medium was collected and cytokine levels quantified by ELISA (BD Biosciences) according to the manufacturer's instructions.

QUANTIFICATION AND STATISTICAL ANALYSIS

We have included number of replicates (equal to number of different donors), statistical tests, and significance criteria in figure legends and in the main text of the manuscript.

Statistical analysis was performed in Excel. We used the paired t test to determine significant differences. Following P values were considered as significant: ***P value $\leq$ *0.001,* ***P*-value $\leq$ 0.01, **P*-value $\leq$ 0.1

DATA AND CODE AVAILABILITY

This study did not generate any datasets.

Cell Reports, Volume *30*

Supplemental Information

TLR4-Mediated Pathway Triggers Interferon-Independent G0 Arrest and Antiviral SAMHD1 Activity in Macrophages

Petra Mlcochova, Helena Winstone, Lorena Zuliani-Alvarez, and Ravindra K. Gupta

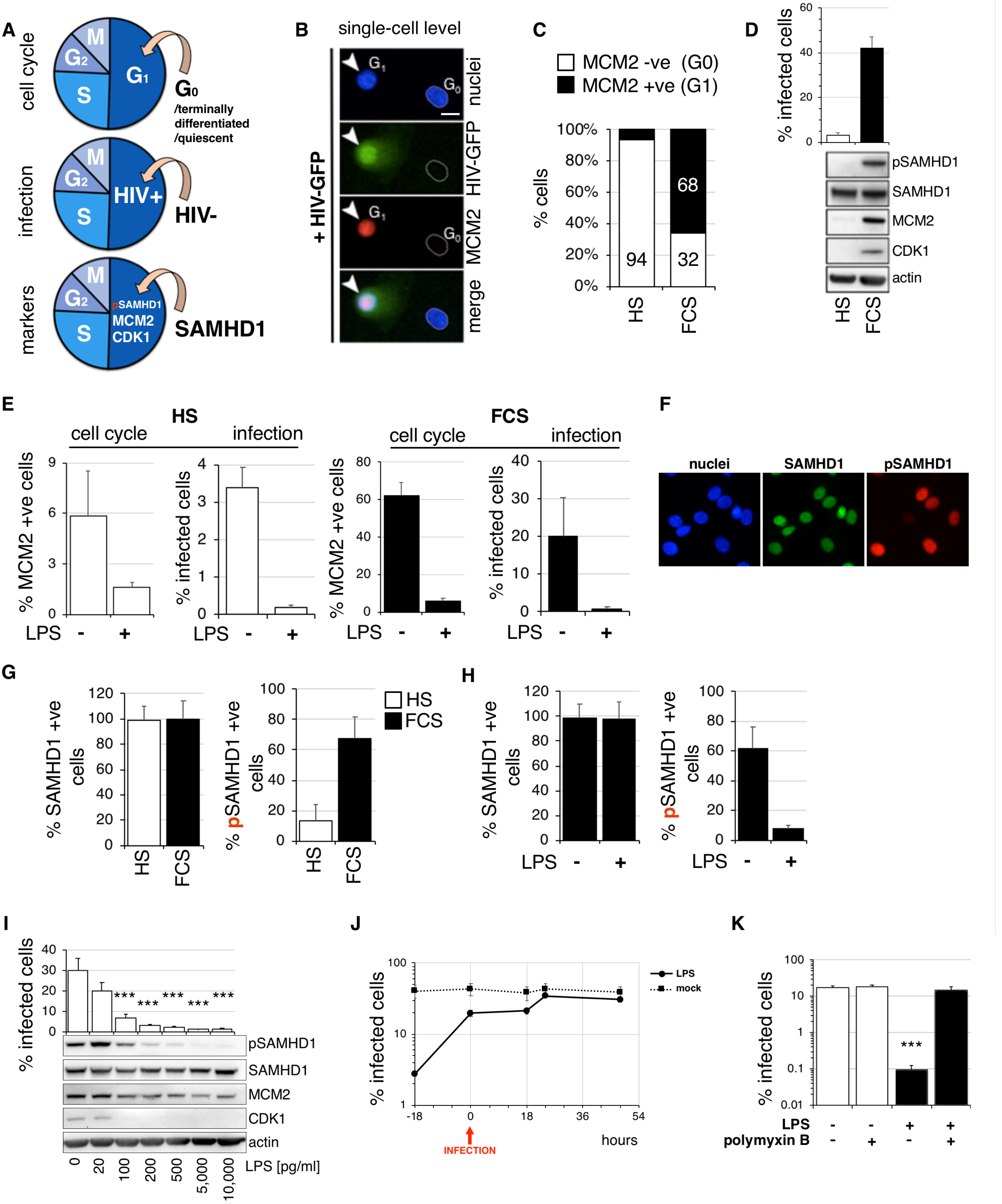
A
cell cycle
infection
markers
M
G2
G1
S
G0
/terminally differentiated /quiescent
HIV+
HIV-
pSAMHD1
MCM2
CDK1
SAMHD1
B
single-cell level
+ HIV-GFP
nuclei
HIV-GFP
MCM2
merge
C
MCM2 -ve (G0)
MCM2 +ve (G1)
% cells
94
32
68
HS
FCS
D
% infected cells
pSAMHD1
SAMHD1
MCM2
CDK1
actin
E
HS
FCS
cell cycle
infection
% MCM2 +ve cells
% infected cells
LPS - +
F
nuclei
SAMHD1
pSAMHD1
G
% SAMHD1 +ve cells
% pSAMHD1 +ve cells
HS
FCS
H
I
LPS [pg/ml]
J
LPS
mock
INFECTION
hours
K
LPS
polymyxin B
***

Figure S1: HS and FCS differentiation protocol used to study TLR4 mediated block to HIV-1 infection. Related to Figure 1-4. **(A)** Diagram of G_0 to G_1 transition in MDM based on our previous study in EMBO J (Mlcochova et al., 2017). MDM in G_0 are quiescent cells where SAMHD1 is dephosphorylated/active. G_0 cells are not permissive to HIV-1 infection. MDM after transition into G_1 phase are positive for MCM2 and CDK1, markers of cell cycle re-entry and G_1 phase. SAMHD1 is phosphorylated/inactive in G_1 cells and MDM are permissive to HIV-1 infection. **(B)** Example of single-cell analysis showing MDM in G_0 (negative for MCM2, non-infected) and G_1 phase (positive for MCM2, HIV-1 infected). In our experiments on average 10^4 cells are recorded and analysed using automated cell-imaging system Hermes WiScan and ImageJ. Scale bars, 20µm. **(C,D)** Quantification of cells in G_0 and G_1 phase. By simple culturing condition we can keep MDM in G_0 (by using Human Serum, HS) or induce G_0 to G_1 transition (by using Fetal Calf Serum, FCS). Quantification can be achieved at single cell level **(B,C)** or from cell lysates using immunoblot **(D)**. **(E)** Cells cultured in HS (MDM mostly in G_0) or FCS (MDM mostly in G_1) were treated with LPS and infected by VSV-G pseudotyped HIV-1 18h later. The percentage of infected and MCM2 positive cells were determined 48h post-infection. (n = 3, mean ± s.e.m.; ***P-value≤0.001, **P-value ≤ 0.01, paired t-test). **(F-H)** Detection of pSAMHD1 at single-cell level. In our experiments on average 10^4 cells are recorded and analysed using automated cell-imaging system Hermes WiScan and ImageJ. **(F)** Representative example of immunofluorescence staining of MDM for total SAMHD1 and phosphorylated SAMHD1. Scale bars, 20µm. **(G)** Quantification of SAMHD1 and pSAMHD1 expression in HS and FCS cultured macrophages. **(H)** Quantification of SAMHD1 and pSAMHD1 expression in FCS cultured macrophages 18h after LPS treatment. **(I-K)** Cells were infected by VSV-G pseudotyped HIV-1 and the percentage of infected cells was determined 48h post-infection. (n=3, mean±SEM; ***P-value≤0.001, paired t-test). **(I)** MDM were treated with increasing concentration of LPS 18h before infection. Cells from a representative donor were used for immunoblotting. **(J)** MDM were treated with LPS at different timepoints starting 18h before infection, at the time of infection and after infection. **(K)** MDM were treated with LPS +/- polymyxin B that blocks the biological effect of LPS.

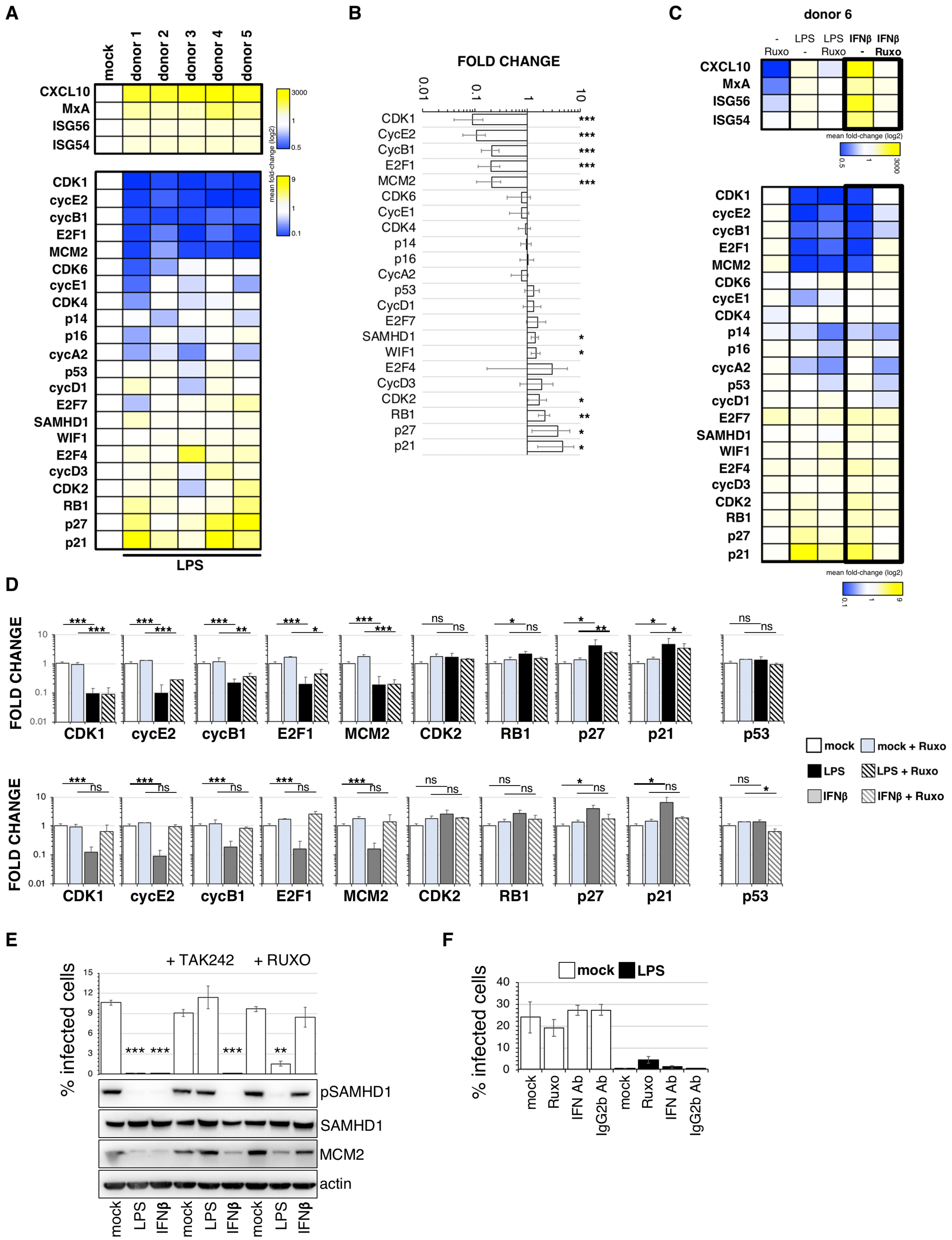
A
mock
donor 1
donor 2
donor 3
donor 4
donor 5
CXCL10
MxA
ISG56
ISG54
CDK1
cycE2
cycB1
E2F1
MCM2
CDK6
cycE1
CDK4
p14
p16
cycA2
p53
cycD1
E2F7
SAMHD1
WIF1
E2F4
cycD3
CDK2
RB1
p27
p21
LPS
mean fold-change (log2)
B
FOLD CHANGE
C
donor 6
D
FOLD CHANGE
mock
mock + Ruxo
LPS
LPS + Ruxo
IFNβ
IFNβ + Ruxo
E
+ TAK242
+ RUXO
% infected cells
pSAMHD1
SAMHD1
MCM2
actin
F
mock
LPS
% infected cells
Ruxo
IFN Ab
IgG2b Ab

Figure S2: IFN-independent and dependent G0 arrest in human MDM. Related to Figure 3. (**A)** A heat map depicting differential gene expression patterns of cell cycle associated transcripts in MDM from 5 different donors treated with LPS for 18h. The colour scale bar corresponds to log-fold expression. (**B)** Relative expression levels (fold change) of cell cycle associated transcripts. (*n* = 6, mean ± SEM; ***P-value≤*0.001,* ***P*-value ≤ 0.01, **P*-value ≤ 0.1, paired *t*-test). (**C)** A heat map depicting differential gene expression patterns of cell cycle associated transcripts in MDM treated with LPS or IFN**β** in the presence or absence of RUXO. The colour scale bar corresponds to log-fold expression. **(D)** Relative expression levels (fold changes) of statistically significantly changed cell cycle associated transcripts after LPS or IFN**β** treatment in the presence or absence of RUXO. (*n* = 4, mean ± s.e.m.; ***P-value≤*0.001,* ***P*-value ≤ 0.01, **P*-value ≤ 0.1, (ns) non-significant, paired *t*-test). **(E)** MDM were treated with TAK242 and RUXO 6h before addition of LPS and interferon b (IFN**β**). Cells were infected by VSV-G pseudotyped HIV-1 18h later. The percentage of infected cells was determined 48h post-infection. (*n* = 3, mean ± s.e.m.; ***P-value≤*0.001,* ***P*-value ≤ 0.01, paired *t*-test). Cells from a representative donor were used for immunoblotting. (**F)** MDM were exposed to anti-IFN Ab/IgG2b non-specific Ab and treated with LPS. Cells were infected by VSV-G pseudotyped HIV-1 18h later. The percentage of infected cells was determined 48h post-infection. (*n* = 3, mean ± s.e.m.; ****P*-value ≤ 0.01, paired *t*-test).

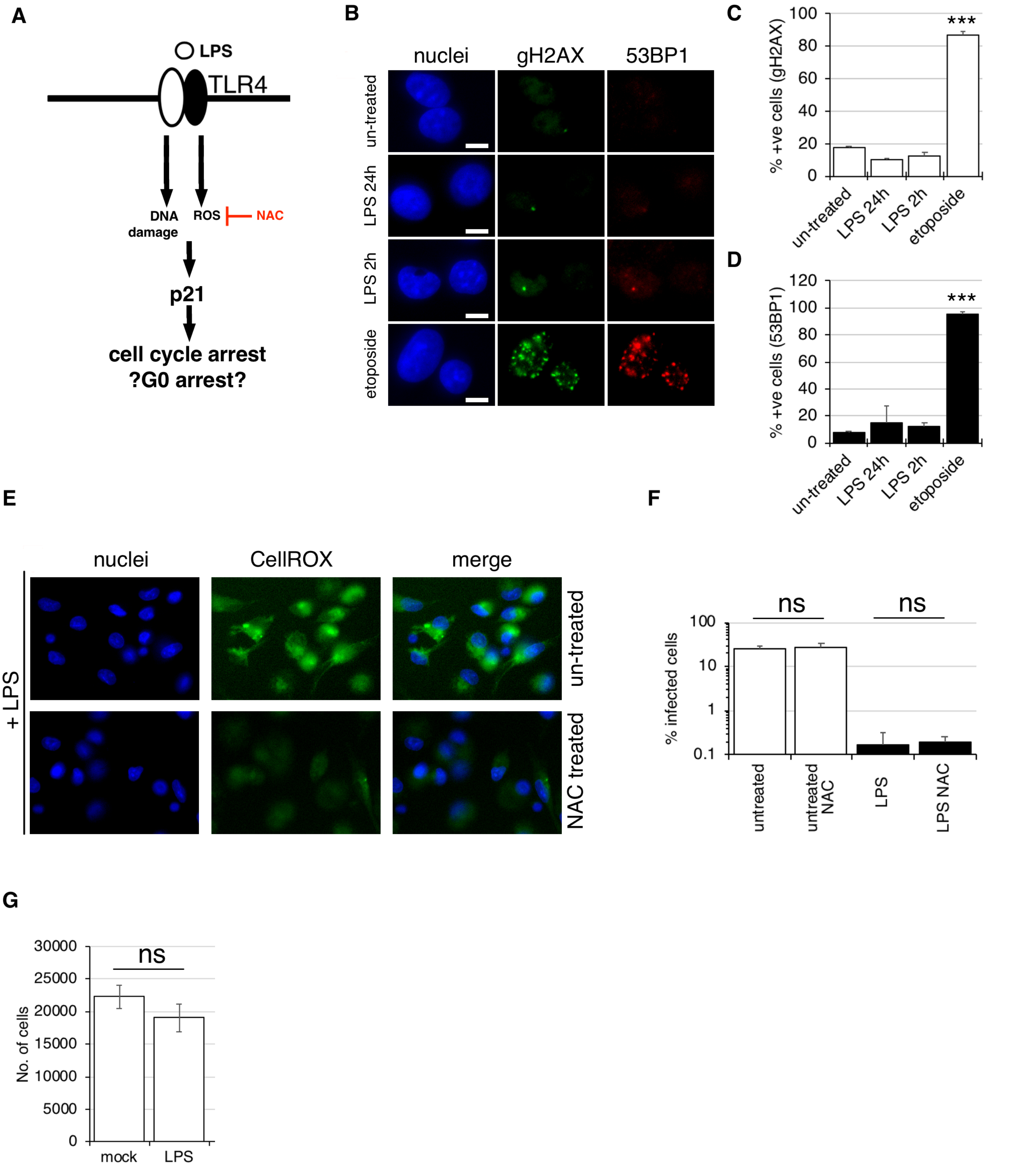

Figure S3: DNA damage and ROS during LPS mediated TLR4 activation in human macrophages. Related to Figure 3. (A) Simplified diagram of LPS activation triggering oxidative stress (ROS production) and DNA damage. ROS inhibitor N-acetyl-L-cysteine (NAC). **(B)** MDM were treated with LPS for 2 or 24h, or with Etoposide for 2h (positive control for DNA damage). Cells were fixed and stained for DNA damage foci positive for H2AX and 53BP1. **(C,D)** Quantification of **(C)** H2AX or **(D)** 53BP1 positive cells. 1,000 cells were acquired using Hermes WiScan automated cell-imaging system (IDEA Bio-Medical Ltd. Rehovot, Israel) and analysed using MetaMorph and ImageJ software. ($n = 3$, mean ± SEM). ***P-value ≤ 0.01, paired *t*-test. **(E)** MDM were treated with LPS in the presence or absence of ROS inhibitor NAC and labelled with CellROX to detect ROS. **(F)** MDM were treated with LPS in the presence or absence of ROS inhibitor NAC 18h before infection. Cells were infected by VSV-G pseudotyped HIV-1 and % infected cells were determined 48h post-infection. ($n = 3$, mean ± SEM). (ns) non-significant, paired *t*-test. **(G)** MDM treated or untreated with LPS were stained for nuclei using DAPI stain. Cell numbers were quantified using Hermes WiScan automated cell-imaging system (IDEA Bio-Medical Ltd. Rehovot, Israel), MetaMorph and ImageJ software ($n = 7$, mean ± SEM). (ns) non-significant, paired *t*-test.

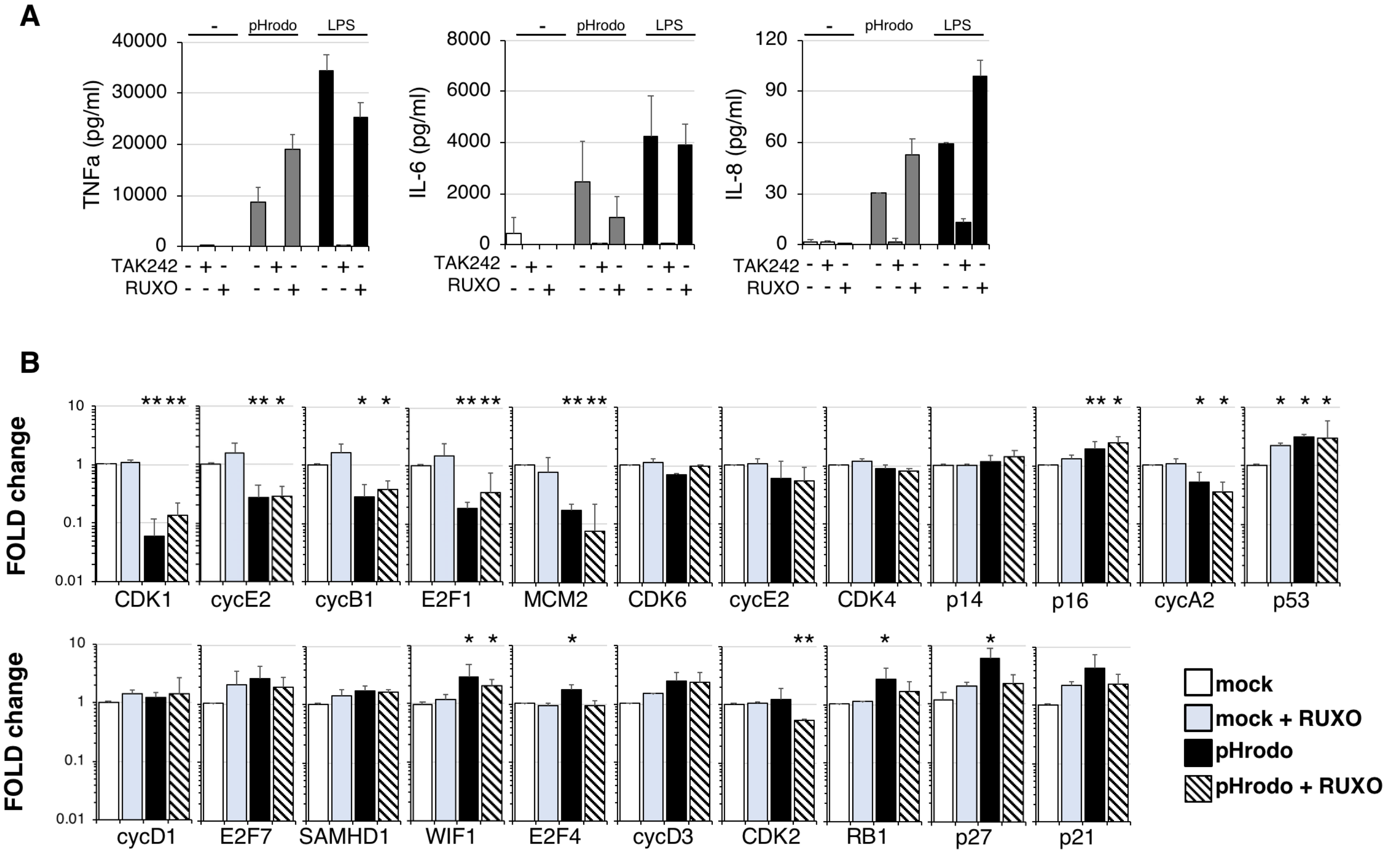

Figure S4: TLR4 activation by gram-negative bacteria. Related to Figure 4. A) MDM were treated with pHrodo E.coli or LPS in the presence or absence of RUXO and cytokines were measured in culture media 24h later. **B)** Relative expression levels (fold changes) of full panel of cell cycle associated transcripts. MDM were treated with RUXO 6h before addition of pHrodo E.coli. Cells were collected 24h later. (*n* = 3, mean ± SEM). **P-value ≤ 0.01, *P-value ≤ 0.1, paired t-test.
